# Reduced EIF6 dosage attenuates TP53 activation in models of Shwachman-Diamond syndrome

**DOI:** 10.1172/JCI187778

**Published:** 2025-02-18

**Authors:** Usua Oyarbide, Valentino Bezzerri, Morgan Staton, Christian Boni, Arish Shah, Marco Cipolli, Eliezer Calo, Seth J. Corey

**Affiliations:** 1 Departments of Pediatrics and Cancer Biology, Cleveland Clinic, Cleveland, Ohio, USA.; 2 Cystic Fibrosis Center, Azienda Ospedaliera Universitaria Integrata, Verona, Italy.; 3 Department of Life Sciences, Health and Health Professions, Link Campus University, Rome, Italy.; 4 Department of Biology and David H. Koch Institute for Integrative Cancer Research, Massachusetts Institute of Technology, Cambridge, Massachusetts, USA.

**Keywords:** Genetics, Hematology, Oncology, Bone marrow, Leukemias, Neutrophils

## Abstract

Shwachman-Diamond syndrome (SDS) is characterized by neutropenia, exocrine pancreatic insufficiency, and bony abnormalities with an increased risk of myeloid neoplasia. Almost all cases of SDS result from biallelic mutations in Shwachman-Bodian-Diamond syndrome (SBDS). SBDS interacts with elongation factor–like 1 (EFL1) to displace eukaryotic initiation factor 6 (EIF6) from the 60S ribosomal subunit. Released EIF6 permits the assembly of ribosomal large and small subunits in the cytoplasm. Decreased EIF6 levels due to haploinsufficiency or missense mutations, which lead to decreased protein expression, may provide a somatic genetic rescue and antileukemic effects. We observed accumulation of EIF6 protein in *sbds*-KO zebrafish models, confirmed this accumulation in patient-derived tissues, and correlated these with changes in ribosomal proteins and tumor protein p53 (TP53) pathways. The mechanism of action for this adaptive response is unknown. To address this, we generated *eif6*-KO zebrafish, which do not survive more than 10 days after fertilization. We also created 2 mutants with low Eif6 expression, i.e., 5%–25% of WT levels, that could survive until adulthood. We bred them with *sbds*-null strains and analyzed their phenotype and biochemical properties. Low Eif6 levels reduced Tp53 pathway activation but did not rescue neutropenia in Sbds-deficient zebrafish. Further studies elucidating the interplay between SBDS, EIF6, and TP53 and cellular stress responses offer promising insights into SDS pathogenesis, somatic genetic rescue, and therapeutic strategies.

## Abstract 2

SDS is a bone marrow failure ribosomopathy. Reduction of ribosomal factor EIF6 provided somatic genetic rescue by attentuating TP53 activation in zebrafish and patient tissue.

## Introduction

Shwachman-Diamond syndrome (SDS) is classically characterized by neutropenia, exocrine pancreatic insufficiency, and bony abnormalities. Patients with SDS also carry an approximately 500-fold increased risk of developing myelodysplastic syndrome (MDS) and/or acute myeloid leukemia (AML) (1). Approximately 90% of SDS cases are caused by biallelic mutations in the Shwachman-Bodian-Diamond syndrome (*SBDS*) gene, which encodes a ribosome maturation factor (2). To our knowledge, there have been no reports of patients with homozygous mutations causing complete loss of SBDS, suggesting that total loss of SBDS protein may be lethal. Complete loss due to homozygous gene ablation results in early embryonic lethality in mice (3) or limited survival in zebrafish larvae (4). Recently, mutations in 3 additional genes, *DNAJC21*, *EFL1*, and *SRP54*, have been identified as causative factors for similar phenotypes (5). A common feature among SBDS, EFL1, DNAJC21, and SRP54 is their involvement in ribosome biogenesis or early protein synthesis.

Ribosome biogenesis begins in the nucleolus with the synthesis of rRNA species and the incorporation of ribosomal proteins for the eukaryotic pre-40S and pre-60S subunits. Both subunits traffic to the cytoplasm, where they undergo the final maturation steps to form a functional 80S ribosome (6). To accomplish this, the pre-60S subunit must release the eukaryotic initiation factor 6 (EIF6), an anti-assembly factor. This anti-association factor prevents the formation of 80S monosomes and polysomes. A critical step is the regulated release of EIF6 from the pre-60S subunit that promotes ribosome assembly in the cytoplasm (7–9). SBDS interacts with the GTPase elongation factor–like 1 (EFL1) to dissociate EIF6 from the cytoplasmic 60S ribosomal subunit (2, 8, 10–12).

Increased expression or mutation of the tumor suppressor tumor protein p53 (*TP53*) gene is frequently found in patients with SDS (13). Studies utilizing murine and zebrafish models have demonstrated that ablation of *Sbds* also leads to the induction of Tp53 and is accompanied by developmental abnormalities and tissue-specific defects (14–17). Cellular studies employing knockdown approaches have corroborated the observations from murine and zebrafish models, showing elevated protein levels of Tp53 and upregulation of its target genes in Sbds-deficient cells (18). *TP53* mutations have been identified in patients with SDS without MDS or AML (19–21), suggesting that a strong selection pressure exists first for cell survival. While monoallelic *TP53* variants may be present in up to 50% of patients with SDS and may persist for years without progression (19), biallelic mutations in *TP53* herald malignant transformation (22). SBDS deficiency is also frequently associated with acquired interstitial deletion of chromosome 20q, where the *EIF6* gene resides, or truncating/missense mutations of *EIF6* in hematopoietic cells (22, 23). Of note, this recurrent del(20q) abnormality has been related to a lower risk for malignancy (24). Considered somatic genetic rescue, the somatic heterozygous mutations in *EIF6* and *TP53* occur in patients with SDS (22). Single SBDS-deficient cells that harbor *EIF6* or *TP53* somatic mutations are mutually exclusive. We hypothesized that *EIF6* mutations that lead to decreased protein levels cause a functional compensation for the germline deficiency by alleviating the SDS ribosome assembly defect, improving translation, and reducing TP53 activation. In contrast, *TP53* mutations can cause cell-cycle arrest, but ribosome assembly defects persist. Partial loss of EIF6 could serve as an adaptive response to reduce the risk of MDS and AML. (However, other clones harboring biallelic mutations in TP53 in the same individual can lead to transformation to myeloid malignancy.) In vitro studies showed that point mutations in *Eif6* rescue the phenotypes of both Efl1 and Sbds deletions (8, 11, 25). These findings were recently strengthened by an in vivo study reporting that *eif6* missense mutations leading to reduced eif6 dosage or its binding to the large 60S ribosomal subunit restore ribosome biogenesis in *sbds*-mutated *Drosophila* models, rescuing the larval lethality observed in *sbds*-mutant flies (26).

To address the mechanism of adaptive rescue by haploinsufficiency of EIF6, we generated *eif6* zebrafish mutants, characterized their phenotype, and analyzed the *sbds*-null fish bred with *eif6* mutants. Eif6-KO larvae did not survive beyond 7–10 days post fertilization (dpf), a shorter survival than *sbds*-KO larvae . Heterozygous expression of Eif6 extended the survival of *sbds^–/–^* fish by approximately 15% and correlated with decreased Tp53 activation. Of note, we validated these results using patient-derived cells. Our studies identify a mechanism for somatic genetic rescue that involves mitigation of TP53 pathways.

## Results

### EIF6 accumulates in the cells of patients with SDS and sbds-null zebrafish.

**Figure 1:**
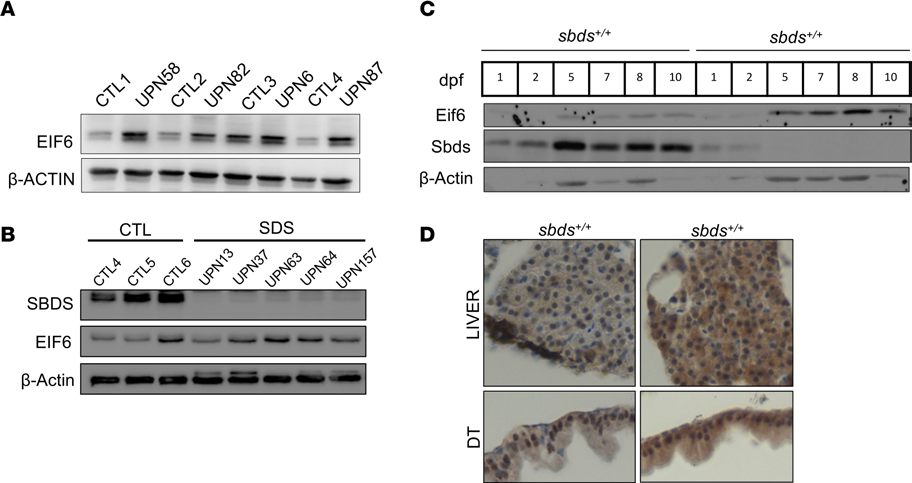
Eif6 accumulation in patients with SDS and *sbds*-KO zebrafish. Western blots showing EIF6 accumulation in SDS lymphoblasts (**A**), SDS PBMCs (**B**), and *sbds*-KO fish from 5 dpf to 10 dpf (**C**). CTL1, healthy control no. 1. (**D**) IHC images of Eif6 expression showing accumulation in the liver and digestive tract (DT) of 10 dpf zebrafish. Original magnification, ×40.

Somatic mutations in *EIF6* or its haploinsufficiency due to loss of 20q have been found in two-thirds of patients with SDS who have a decreased risk of developing myeloid malignancies (22, 23). To investigate the effect of EIF6 on the pathophysiology of SDS, we first observed an accumulation of EIF6 in lymphoblasts and PBMCs from individuals with SDS compared with controls (Figure 1, A and B, and Supplemental Table 2; supplemental material available online with this article; https://doi.org/10.1172/JCI187778DS1). Previously, we reported increased Eif6 protein levels in *sbds*-null zebrafish larvae at 10 dpf (4). Here, we discovered that EIF6 accumulation occurred at earlier stages of embryonic development (Figure 1C). Eif6 accumulation occurred as early as 5 dpf in *sbds^–/–^* zebrafish, which coincided with depletion of Sbds protein. Next, we determined the location of this accumulation by larvae at 10 dpf. We performed IHC, which detected a cytoplasmic accumulation of Eif6 in *sbds*-null fish compared with their WT siblings (Figure 1D).

### EIF6 is essential for zebrafish embryonic development.

**Figure 2:**
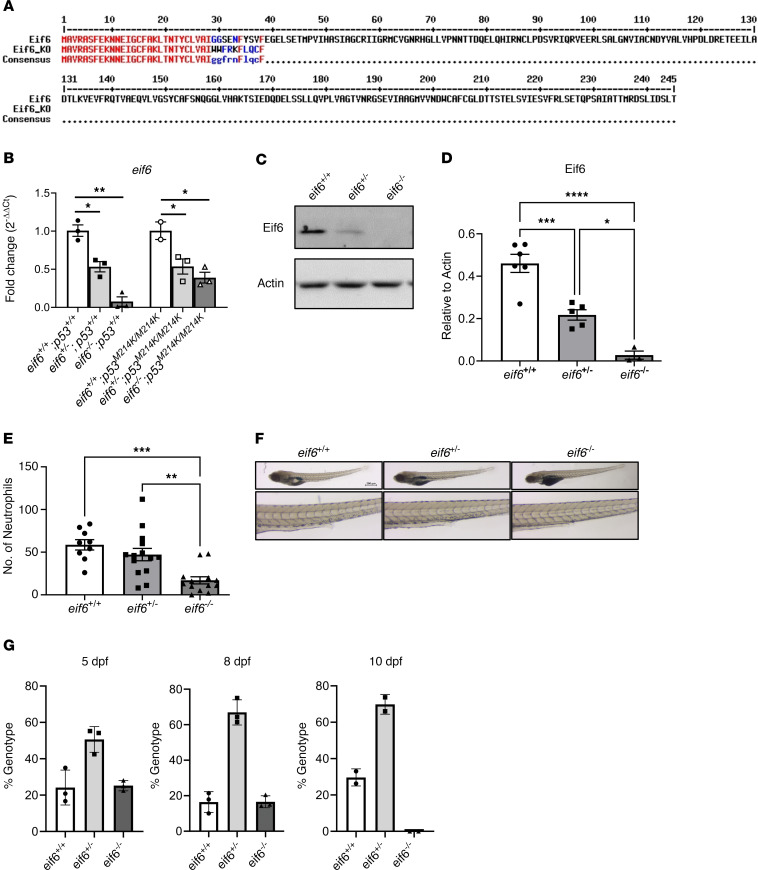
Eif6 is essential for zebrafish embryonic development. (**A**) Amino acid alignment of WT and *eif6* KO zebrafish; the insertion of 1 bp causes early truncation. (**B**) mRNA *eif6* levels by RT-qPCR on the *tp53^+/+^* and *tp53^M214K/M214K^* backgrounds at 5 dpf. (**C**) Western blots showing the absence of Eif6 protein in the *eif6^–/–^* zebrafish larvae at 5 dpf. Note the dose effect in the *eif6^+/–^* fish compared with their WT siblings. (**D**) Western blot quantification. (**E**) *eif6* KO showed a significantly lower number of neutrophils than the WT siblings at 5 dpf. (**F**) Sudan black staining for neutrophils counts (original magnification, ×2.5 and ×8). (**G**) Survival percentages for *eif6^+/+^, eif6^+/–^*, and *eif6^–/–^* siblings at 5, 8, and 10 dpf. *P < 0.05, **P < 0.01, and ***P < 0.001, by ANOVA. Data represent the mean ± SEM.

We hypothesized that the accumulation of EIF6 contributes to the pathogenesis of SDS. To address this, we used CRISPR/Cas9 editing to create an *eif6*-mutant allele with a 1 bp deletion (*eif6^lri110^*, hereafter denoted as *eif6^–^*) that produced a premature termination codon (Figure 2A). Reverse transcription quantitative PCR (RT-qPCR) revealed that *eif6* transcripts were significantly decreased in the *eif6^+/–^* and *eif6^–/–^* strain compared with WT siblings (Figure 2B). Western blotting revealed an absence of Eif6 expression at 5 dpf in the *eif6^–/–^* fish, and the heterozygotes showed half the amount of protein compared with their WT siblings (Figure 2, C and D). By 5 dpf, they showed severe defects, such as cardiac edema, failure of the swim bladder to inflate, a smaller head and eyes, and a decreased number of neutrophils compared with their *eif6^+/–^* and *eif6^+/+^* clutchmates (Figure 2, E and F). No *eif6^–/–^* fish survived beyond 10 dpf (Figure 2G).

RNA-Seq was performed on whole zebrafish larvae from *eif6^+/+^* and *eif6^–/–^* at 5 dpf. Hierarchical clustering showed a high overlap between the replicates within each genotype while clearly separating the triplicates into 2 groups. RNA-Seq analysis at 5 dpf larvae revealed 1557 upregulated and 668 downregulated genes in mutants. To gain deeper understanding of the molecular processes and signaling pathways affected in *eif6^–/–^*, Kyoto Encyclopedia of Genes and Genomes (KEGG) enrichment analysis was conducted on genes that were either upregulated or downregulated in *eif6*-null fish. The analysis revealed that differentially expressed genes (DEGs) were enriched in a variety of biological processes and signaling pathways. Specifically, these included ribosome and oxidative phosphorylation pathways, highlighting their marked involvement in the genetic alterations observed in the *eif6*-null phenotype (Table 1).

### Low levels of Eif6 are sufficient for survival.

**Figure 3:**
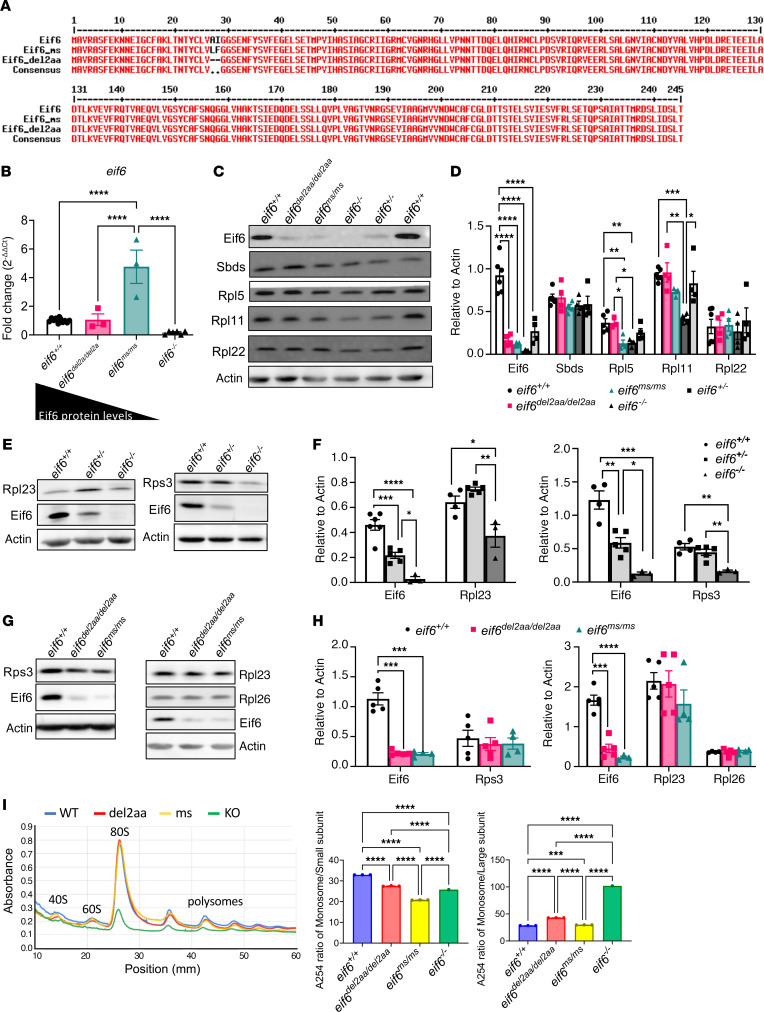
Low levels of Eif6 are enough for survival to adulthood, but only an absence of *eif6* KO affects the levels of ribosomal proteins. (**A**) Amino acid alignment of the 2 *eif6* mutants (*eif6^ms/ms^* and *eif6^del2aa/del2aa^*) compared with the Eif6 WT sequence. (**B**) mRNA levels in all 3 *eif6* mutants and WT zebrafish at 5 dpf. (**C**) Western blots showing Eif6, Sbds, and RP levels in the different *eif6* mutants. (**D**) Western blot quantification. (**E**) Western blots showing Rpl23 and Rps3 in *eif6^+/+^*, *eif6^+/–^*, and *eif6^–/–^* siblings at 5 dpf. (**F**) Western blot quantification relative to actin. (**G**) Western blotting showing Rpl23 and Rps3 expression in *eif6^+/+^*, *eif6^del2aa/del2aa^*, and *eif6^ms/ms^* zebrafish at 5 dpf. (**H**) Western blot quantification relative to actin. (**I**) Polysome profiles of *eif6* WT and mutants at 5 dpf. *P < 0.05, **P < 0.01, and ***P < 0.001, by 1-way ANOVA. Data represent the mean ± SEM.

When targeting *eif6* exon 2 to create the *eif6*-null fish analyzed above, we obtained 2 other founders with different mutations, 1 with a deletion of 6 bp in-frame that caused a deletion of 2 amino acids (*eif6^lri111^*, hereafter denoted as *eif6^del2aa^*) and another with 4 bp in-frame missense mutation that resulted in a change in 2 amino acids (*eif6^lri112^*, hereafter denoted as *eif6^ms^*) (Figure 3A). Surprisingly, both mutants survived to adulthood and were fertile, allowing us to create maternal zygotic (MZ) lines. We evaluated the *eif6* mRNA and protein levels of these *eif6*-mutant lines. At 5 dpf, mRNA levels of the *eif6^del2aa/del2aa^* mutant were similar to those of the WT fish, but in the *eif6^ms/ms^* mutant, the mRNA levels were upregulated 4-fold (Figure 3B). We next determined protein expression levels in each mutant strain at 5 dpf. The *eif6^del2aa/del2aa^* mutant made 20%–30% and the *eif6^ms/ms^* mutant made 5%–10% of the protein expressed by the WT fish (Figure 3, C and D). Decreased protein levels of mutant EIF6 were reported in human tissues (22, 23). Because there was no evidence for transcriptional repression, these results suggested that mutant Eif6 caused protein instability (see below), degradation, or translational inefficiency.

Since there were decreased ribosomal protein transcripts in the *eif6* KO (Figure 3, A and B), we explored how the different *eif6* mutations affect Sbds and other ribosomal proteins. Sbds protein levels were not affected in any of the 3 mutants (*eif6^del2aa/del2aa^*, *eif6^ms/ms^*, and *eif6^–/–^*) compared with WT at 5 dpf (Figure 3C). Rps3 and Rpl23 were decreased in the *eif6^–/–^* mutants. Eif6 interacts with Rpl23, located close to the surface of the 60S subunit (27). Rpl5 and Rpl11 can activate the tumor suppressor protein Tp53 pathway by binding to Mdm2 (28). This process is a key component in the context of ribosomal stress. Additionally, somatic mutations in RPL5 and RPL22 have been identified in patients with SDS. This suggests that mutations in regulators of the nucleolar signaling pathway, like RPL5 and RPL22, may interfere with nucleolar stress–induced stabilization of TP53 (29). Interestingly, Rpl5 was significantly reduced in the *eif6^–/–^* and *eif6^ms/ms^* mutants, the *eif6*-KO, and the lowest Eif6 protein mutant respectively, and Rpl11 only in the *eif6*-KO mutant (Figure 3, C–H). Rpl22 did not changed in any of the mutants. Thus, Eif6 levels affected the expression of other ribosomal proteins. Altogether, the changes in Rpl23, Rpl5, Rpl11, and Rps3 levels suggested more global effects due to loss of Eif6 and merit further investigation.

We performed polysome profiling to study how alterations in Eif6 levels might affect translation. Polysome profiles showed a significant reduction in the monosomal and 40S ribosomal subunits ratio between *eif6^+/+^* and all *eif6* mutants at 5 dpf. Interestingly, loss of Eif6 did not result in any free ribosomal subunits and showed a significant reduction in the levels of 80S ribosomes, while *eif6^del2aa/del2aa^* and *eif6^ms/ms^* mutants showed a polysome profile similar to that of the *eif6* WT (Figure 3I). In summary, the absence of Eif6 led to aberrant ribosome structures, which could explain the early lethality observed in the *eif6*-KO zebrafish line. These changes led to an earlier demise than the loss of Sbds (4).

### Tp53 pathway activation in eif6 mutants does not affect survival rates.

**Figure 4:**
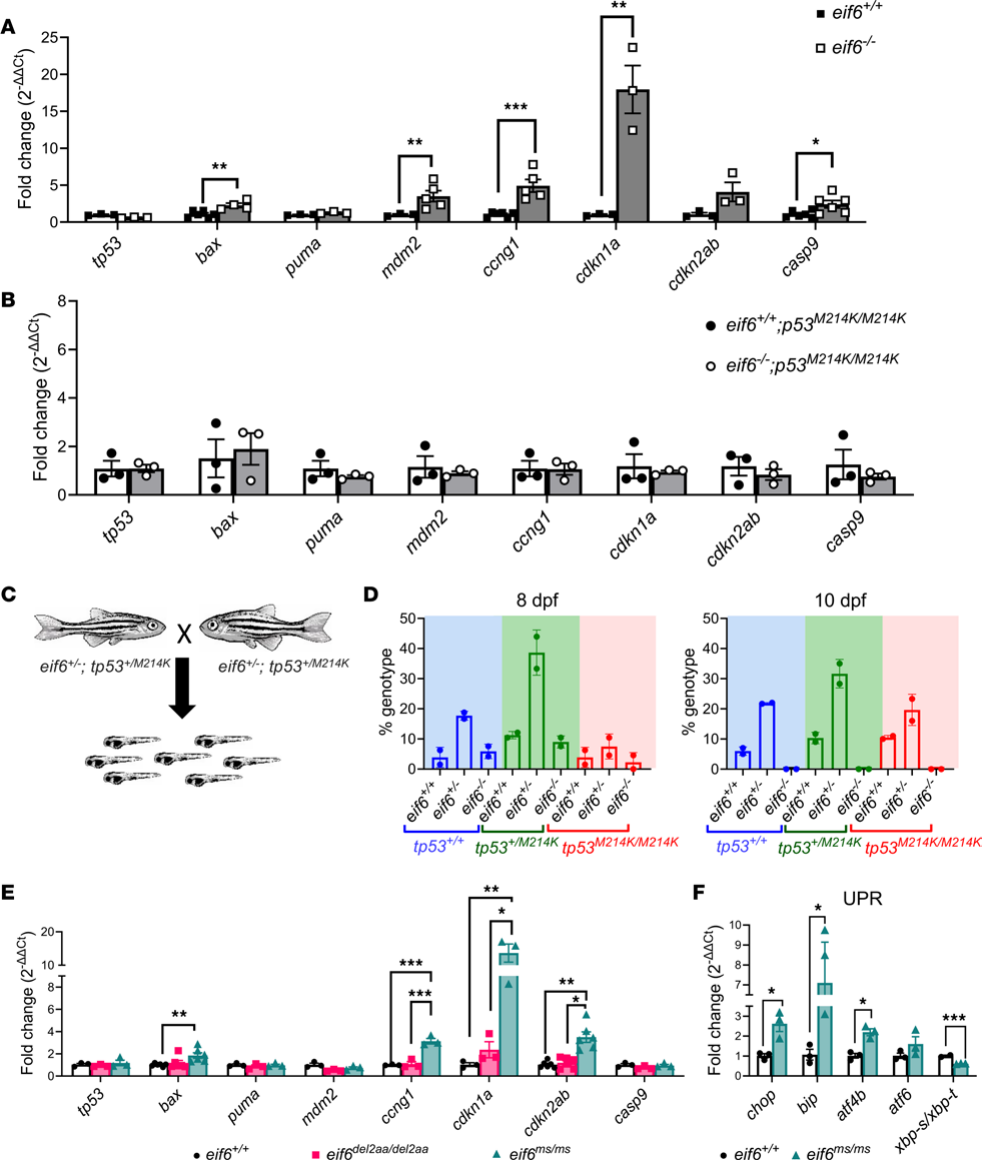
Tp53 pathway activation in *eif6* mutants does not affect survival rates. RT-qPCR analysis of *tp53* and its targets of WT and *eif6* KO on the (**A**) *tp53^+/+^* and (**B**) *tp53^M214K/M214K^* backgrounds. (**C**) Scheme of the double-heterozygote crosses (*eif6^+/–^* *tp53^+/M214K^*). (**D**) Percentage of survival for double-heterozygote crosses of *eif6^+/–^* *tp53^+/M214K^* at 8 and 15 dpf. (**E**) RT-qPCR analysis of *tp53* and its targets (*P < 0.05, **P < 0.01, and ***P < 0.001, by 1-way ANOVA), and (**F**) UPR markers in WT and *eif6^ms/ms^* zebrafish at 5 dpf (*P < 0.05 and ***P < 0.001, by 2-tailed *t* test). Data represent the mean ± SEM.

We previously reported that deletion of Sbds in zebrafish caused activation of the Tp53 pathways involving *tp53*, *cdkn1a*, *mdm2*, *cdkn2ab*, *bax*, *puma*, and *ccng1* (4). Since Sbds and Eif6 are part of the same ribosomal assembly pathway, we hypothesized that these pathways were also affected in the *eif6* KO at 5 dpf. Our results demonstrated a significant upregulation of Tp53 targets (*cdkn1a*, *mdm2*, *bax*, *ccng1*, and *casp9*) in the *eif6*-null fish (Figure 4A). To determine whether Tp53 is involved in the dysregulation of its own pathway, we created a new *eif6^–/–^* zebrafish on the *tp53^M214K^* background (*eif6^+/–^* *p53^M214K/M214K^*). When we compared with WT *eif6* versus *eif6^–/–^*, we observed that *eif6* mRNA level differences were similar to those observed on the *tp53* WT background (Figure 2B). As expected, all Tp53 targets previously evaluated (*bax*, *puma*, *mdm2*, *ccng1*, and *casp9*) were not upregulated (Figure 4B). 

To investigate the effect of Tp53 activation on survival rates in the *eif6* mutants, we incrossed double heterozygotes and assessed their progeny at 8 and 10 dpf (Figure 4, C and D). At 8 dpf, all 9 of the predicted genotypes were found at the expected Mendelian ratio. However, at 10 dpf, we did not detect any *eif6^–/–^* zebrafish alive independently of the *tp53* genotype (Figure 4D). Thus, the *tp53^M214K/M214K^* mutants did not rescue the Eif6 deficiency. We recently reported that *tp53^M214K/M214K^* mutants also did not rescue Sbds deficiency (4, 17).

Next, we sought to determine whether the Tp53 pathway was also affected in the *eif6^del2aa/del2aa^* and *eif6^ms/ms^*. Interestingly, we found that the Tp53 targets *cdkn1a*, *cdkn2ab*, and *ccng1* were upregulated only in the *eif6^ms/ms^* mutant. These findings suggested that the quantity of Eif6 protein played a crucial role in regulating Tp53 (Figure 4E). Since we had observed that *eif6* mRNA levels were upregulated in this mutant, we also checked some markers for the unfolded protein response (UPR). The *eif6^ms/ms^* mutants exhibited an upregulation of some UPR markers (*chop*, *bip*, and *atf4b*) (Figure 4F), which could potentially lead to protein degradation and result in the lower levels of Eif6 protein we observed.

### Erythrocyte and neutrophil counts are unaffected in eif6 mutants.

Using Sudan black and O-dianisidine staining, we found that all zebrafish expressing low levels of Eif6 produced neutrophils and RBCs, with no differences compared with WT (Supplemental Figure 1, A and B). While *eif6^–/–^* zebrafish also produced these cells, substantial developmental defects hindered comparisons with their WT siblings.

### Partial rescue of sbds^–/–^ occurs with 1 copy of the WT eif6 allele.

**Figure 5:**
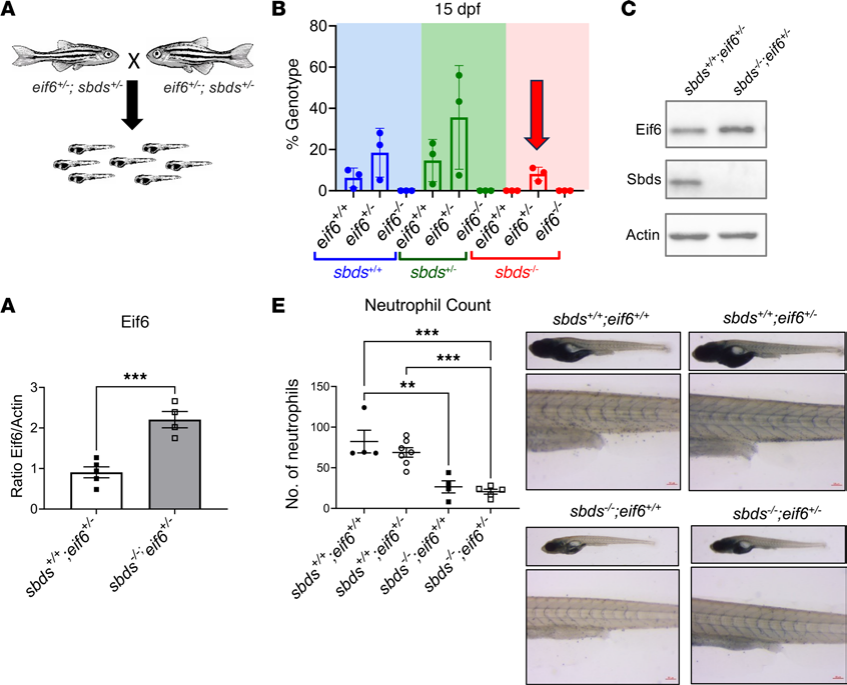
Partial rescue of *sbds* KO with 1 copy of the WT *eif6* allele. (**A**) Scheme showing the incrossing of 2 double-heterozygotes (*eif6^+/–^ sbds^+/–^*). (**B**) Genotype percentages at 15 dpf of an incrossing of double-heterozygotes. (**C**) Western blots showing Eif6 accumulation in *sbds* KO fish with only 1 copy of the *eif6* WT allele (*eif6^+/–^*). (**D**) Western blot quantification of Eif6 protein expression. (**E**) Sudan black staining to detect the number of neutrophils in 10 dpf siblings. Scale bars: 200 μm and 50 μm. Data represent the mean ± SEM.

Since we observed an accumulation of Eif6 protein in the *sbds^–/–^* zebrafish (Figure 1, C–E), we investigated whether reduction of Eif6 would be beneficial for them, as has been suggested in patients with SDS (22, 23). We created double heterozygotes (*eif6^+/–^* *sbds^+/–^*) and assessed survival rates at 15 dpf (Figure 5, A and B). The only *sbds^–/–^* fish that survived were those that harbored 1 copy of WT eif6 (*eif6^+/–^*). This suggested that the Eif6 dosage may be crucial for somatic genetic rescue. However, the *eif6^+/–^ sbds^–/–^* zebrafish did not survive to adulthood. Next, we measured Eif6 protein levels by Western blotting. We detected an accumulation of Eif6 in the *eif6^+/–^* *sbds^–/–^* fish compared with their *eif6^+/–^* *sbds^+/+^* siblings (Figure 5, C and D). This partial rescue in survival indicated that some accumulation of Eif6 could be toxic and that other ribosomal stress responses persist.

We previously reported that *sbds*-null zebrafish have a lower number of neutrophils than do their WT siblings. To determine whether the amount of Eif6 protein affects the neutrophil, we measured the number of neutrophils in zebrafish on the *eif6*-null background. The number of neutrophils was lower on the *sbds^–/–^* background in both *eif6^+/+^* and *eif6^+/–^* zebrafish compared with their *sbds^+/+^* siblings (Figure 5E). Thus, the neutropenia could not be rescued.

### Low Eif6 levels decrease Tp53 pathway activation and partially rescue survival but do not rescue neutropenia in sbds^–/–^ zebrafish.

**Figure 6:**
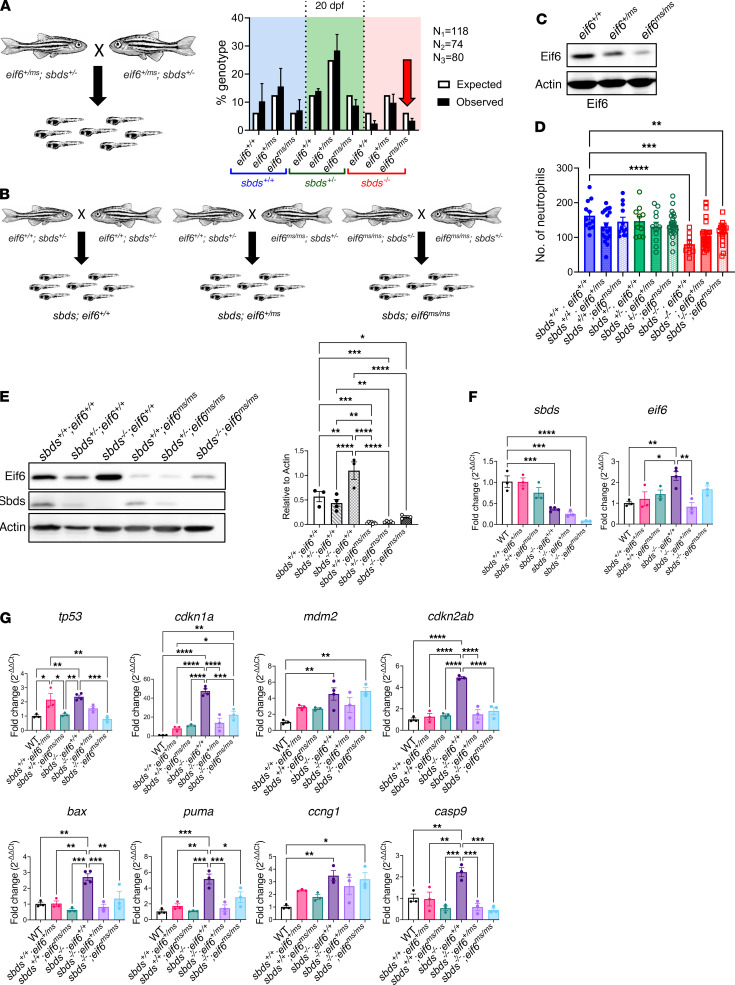
Correlation of Eif6 levels with tp53 activation in *sbds*-KO zebrafish. (**A**) Scheme of *sbds^+/–^* crossings in the WT and Eif6 missense mutants and percentage of genotypes observed at 20 dpf. (**B**) Schemes of the 3 different crosses to obtain *sbds* WT and KO zebrafish on the *eif6^+/+^*, *eif6^+/ms^*, and *eif6^ms/ms^* backgrounds. (**C**) Immunoblot showing Eif6 accumulation in *sbds-*KO zebrafish. (**D**) Neutrophil counts at 10 dpf. *P < 0.05, **P < 0.01, ***P < 0.001, by 1-way ANOVA with Dunnett’s test. (**E**) Protein levels in *sbds^+/+^*, *sbds^+/–^*, and *sbds^–/–^*, in the *eif6^+/+^* and eif6ms/ms mutant backgrounds, along with protein quantification. (**F**) RT-qPCR results for *sbds* and *eif6* (**G**) and the tp53 pathway. *P < 0.05, **P < 0.01, ***P < 0.001, and ****P < 0.0001, by 1-way ANOVA with Tukey’s test. Data represent the mean ± SEM.

We showed that *sbds*-KO fish with only 1 WT *eif6* allele (*eif6^+/–^)* could survive longer (Figure 5A), so we next incrossed double-heterozygotes carrying the *eif6* missense mutation (*sbds^+/–^ eif6^+/ms^*). Similar to our previous observations, we found that *sbds*-KO fish with slightly higher survival rates were those on the *eif6^+/ms^* and *eif6^ms/ms^* backgrounds (Figure 6A).

In a dihybrid cross (*eif6^+/–^ sbds^+/–^*), the expected Mendelian ratio is 1 WT (*sbds^+/+^ eif6^+/+^*) or 1 double-mutant (*sbds^–/–^ eif6^–/–^*) of 16 fish (6.25%). This low frequency makes collecting these specific genotypes challenging, especially for RNA extraction purposes. Unlike the *eif6*-KO fish, the *eif6^ms/ms^* mutants were viable. To further study how low Eif6 levels affect *sbds* KO, we created an *sbds*-mutant line in the *eif6^ms^* mutation (*sbds^+/–^* *eif6^ms/ms^*). In this case, the expected Mendelian ratio is 1 of 4 *sbds* WT/mutant fish on the *eif6^ms/ms^* background (25%). To study how Eif6 levels affect *sbds* KO at 10 dpf, we performed 3 different crosses: (a) incross of *sbds^+/–^* on the *eif6* WT background (*sbds^+/–^* *eif6^+/+^*); (b) incross of *sbds^+/–^* on the *eif6* missense mutation background (*sbds^+/–^* *eif6^ms/ms^*); and (c) outcross of *sbds^+/–^* on the *eif6* WT background (*sbds^+/–^ eif6^+/+^*) with a *sbds^+/–^* on the *eif6* missense mutation background (*sbds^+/–^ eif6^ms/ms^*). With these 3 crosses, we determined differences between *eif6^+/+^*, *eif6^ms/ms^*, and *eif6^+/ms^* in the *sbds* WT and *sbds*-KO fish, respectively (Figure 6B). We then determined Eif6 protein levels in fish on the *sbds* WT background at 5 dpf and found that the protein levels in the heterozygotes were approximately 50% of the levels in WT fish (Figure 6C).

Previously, we demonstrated that *sbds*-KO zebrafish had substantially lower numbers of neutrophils compared with their WT siblings (Figure 4D) (4). At 10 dpf, we determined the number of neutrophils and found that *sbds^–/–^* fish had significantly lower numbers of neutrophils than did the WT fish, regardless of *eif6* genotyping (Figure 6D). These results are consistent with those previously observed in *eif6^+/–^* fish (Figure 5E), suggesting that Eif6 levels did not affect neutrophil counts in our zebrafish models.

Next, we assessed Eif6 levels in *eif6* WT and missense mutants and found an accumulation of Eif6 in fish on the *sbds*-KO backgrounds at 10 dpf. We chose this time point on the basis of our previous studies, which showed that *sbds^–/–^* fish had no detectable Sbds protein at 10 dpf (4). However, this accumulation was significantly lower in *eif6^ms/ms^* fish than in *eif6^+/+^* fish (Figure 6E). Additionally, we analyzed *sbds* and *eif6* mRNA levels across all genotypes and found, as expected, that *sbds* mRNA levels were decreased in *sbds*-KO zebrafish, regardless of the *eif6* background (Figure 6F).

Subsequently, we examined markers of tp53 pathways. Surprisingly, when Eif6 levels were low (*eif6^+/ms^*) or lower (*eif6*^ms/ms^), *sbds*-KO fish did not exhibit a pronounced activation of the Tp53 pathway compared with those on the *eif6* WT background (*eif6^+/+^*), in which *cdkn1a, cdknd2ab, bax,* *puma*, and *casp9* expression levels were strongly upregulated (Figure 6G). Hence, low levels of Eif6 reduced the activation of the tp53 pathway, which could serve to mitigate the role of Eif6 accumulation in TP53-mediated stress responses and apoptosis.

### Tp53 is upregulated in SDS patient–derived cell lines.

**Figure 7:**
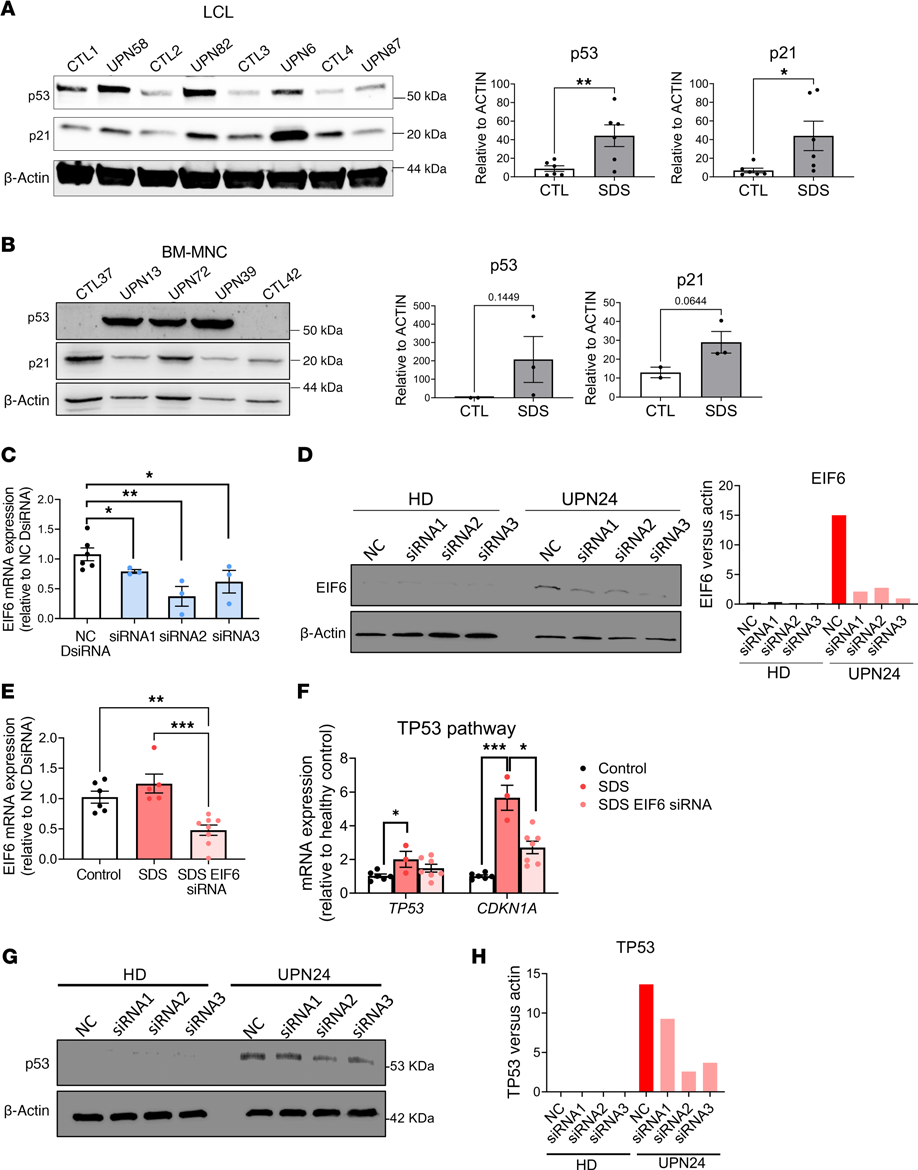
Tp53 activation in SDS patient–derived cells. Western blots and quantification of (**A**) LCLs and (**B**) BM-MNCs. *EIF6* mRNA (**C**) and protein (**D**) levels in 3 different siRNAs. (**E**) *EIF6* mRNA levels in patients with SDS with and without siRNA (**F**) *TP53* and *CDKN1a* mRNA levels are decreased after siRNA in patients with SDS. (**G**) Western blots showing decrease in TP53 levels after EIF6 knockdown using 3 different siRNAs. (**H**) TP53 protein quantification. Data represent the mean ± SEM. HD, healthy donor; NC, negative control.

Because we observed that the Tp53 pathway plays an important role in our zebrafish models of SDS, we sought to determine whether human SDS–derived tissues exhibited the same level of activation. We assessed TP53 and p21 protein levels in SDS patient–derived lymphoblastoid cell lines (LCLs) and bone marrow mononuclear cells (BM-MNCs) and detected a significant 4-fold increase in SDS LCL cells compared with the controls (Figure 7A). We confirmed this finding in primary BM-MNCs freshly isolated from patients with SDS, in which SDS cells had more extensive increases in TP53 levels (Figure 7B).

### Knockdown of EIF6 in SDS-derived LCL decreases TP53 and CDKN1A mRNA levels.

Having observed increased EIF6 protein levels in LCL cells derived from patients with SDS (Figure 1B), we hypothesized that decreased EIF6 levels could mitigate the activation of the TP53 pathway. We first tested 3 different *EIF6* shRNAs (Figure 7, C and D). We chose siRNA no. 2, which showed the highest silencing efficacy, inducing a 5.7-fold reduction in *EIF6* mRNA expression and a 9-fold reduction in protein levels (Figure 7, D and E). Corresponding to the decreased EIF6 protein levels, *TP53* and *CDKN1A* levels in these cells were significantly decreased from approximately 5.6-fold to approximately 2.7-fold (Figure 7F). Additionally, in SDS-deficient cells, TP53 protein levels decreased following the reduction of EIF6. In contrast, TP53 levels in healthy controls remained unaffected (Figure 7, G and H). Hence, low EIF6 levels mitigated the activation of the TP53 pathways, alleviating the cellular stress in SBDS-deficient cells as in *sbds^–/–^* zebrafish and offering a mechanism for somatic cell rescue.

## Discussion

We report here the generation and analysis of *eif6*-null and missense mutations that contributed to pathogenesis in a SDS zebrafish model. Loss of Eif6 resulted in an earlier lethality than with loss of Sbds. However, low levels of Eif6 permitted zebrafish to survive through adulthood and be fertile, suggesting a critical dosage effect. Polysome analysis revealed a flattened profile for *eif6^–/–^* fish, but a normal pattern for hypomorphic Eif6 fish. As in the *sbds^–/–^* model for SDS (4, 17), loss of Tp53 function did not rescue the early lethality of *eif6^–/–^* larvae. Low protein levels of Eif6 conferred a modest increase in survival of *sbds*-deficient fish, which correlated with decreased activation of Tp53 pathways. Altogether, our results indicated that there was a critical Eif6 dosage effect on survival and that additional TP53-independent pathways contributed to larval lethality for both *sbds^–/–^* and *eif6^–/–^* zebrafish.

Kennedy et al. (22), Tan et al. (23), and Machado et al. (29) made notable contributions to our knowledge of the pathophysiology of SDS by identifying competing clones harboring *EIF6* and *TP53* mutations. First suggested by Xia et al. (19), *TP53* mutations arise in an age-dependent manner in individuals with SDS, unlike the absence of these mutations in severe congenital neutropenia. These studies suggested that TP53 and EIF6 afford somatic genetic rescue with different fates. Mutations in *EIF6* lead to improve ribosome joining and translational efficiency (30). Mutations in *TP53* existed as either monoallelic or biallelic, with the risk of MDS/AML greater in biallelic conditions. While monoallelic *TP53* variants might be present for years without progression to malignancy, biallelic mutations often herald a malignant transformation. These clinical studies suggested adaptive (decreased EIF6 levels) or maladaptive (loss of TP53 tumor suppressor activities) responses in SDS. The connection between the two and the characterization of the adaptive mechanism for EIF6’s dosage have been elusive.

The TP53 pathway is activated by ribosome biogenesis defects and aberrant protein translation (31, 32). Sbds deficiency induces activation of the TP53 tumor suppressor pathway and subsequent growth arrest and/or apoptosis (4, 14, 17, 26). To determine the role of EIF6 in SDS pathophysiology and TP53 activation, we used SDS patient-derived cells and detected significantly higher levels of EIF6. This accumulation was previously observed in mouse (8) and zebrafish SDS models (4, 17). Our study further investigated this accumulation phenomenon in a zebrafish *sbds* KO. We found that the accumulation of Eif6 was more prominent in the cytoplasm compared with other cellular compartments. These results may indicate that SBDS deficiency affects the cytoplasmic steps of ribosome biogenesis, where ribosomal subunits mature and assemble before being utilized in protein synthesis. In addition, Jaako et al. demonstrated that transgenic mice overexpressing *Eif6* exhibit a defect in ribosome assembly similar to that observed SDS (26). This observation suggests that an excess of EIF6 can disrupt normal ribosome biogenesis processes, potentially leading to phenotypic manifestations reminiscent of ribosomopathies like SDS. These findings underscore the stoichiometric balance required for ribosome assembly and function, implicating EIF6 dysregulation as a contributing factor in certain genetic disorders affecting bone marrow function. With these observations we wondered whether this accumulation of Eif6 in the *sbds* deficient cells were causing cellular stress that led to Tp53 activation.

Our *eif6* KO showed critical developmental defects and early mortality, similar to what previously was observed in the Eif6-null mouse (26). However, the hypomorphs (low Eif6 expressers mutants) were able to survive until adulthood and were fertile. To study how the amount of Eif6 affects translation activity in our zebrafish models, we performed polysome profiling in all three Eif6 mutants. Only the KO exhibited substantial defects, characterized by the near absence of free 40S and 60S ribosomal subunits and markedly low levels of 80S ribosomes and polysome fractions. In addition, ribosomal proteins were also reduced. This severe disruption indicates a critical impairment in ribosome assembly and function. In summary, *eif6* KOs, lacking Eif6, failed to produce the necessary ribosomal subunits, leading to a cascading failure in protein synthesis machinery. This disruption may lead to the observed critical developmental failures and early death. Interestingly, none of the low Eif6 expressers, showed ribosomal impairment in the polysome profile. This highlights the critical importance of EIF6 dosage, or stoichiometry, in non-diseased cellular function and viability and emphasizes the severe consequences of its total absence on ribosomal integrity and protein production. Only a 10% level of Eif6 is sufficient for normal cellular function, development, and survival. Zebrafish provides a model for developing drugs to modulate Eif6 levels that would ameliorate SDS. Zebrafish can provide an organismal model for validating small molecule modulators that could reduce Eif6 protein levels via transcriptional or post-translational mechanisms and ameliorate SDS. EIF6 dosage is crucial, as low levels of EIF6 protein mitigate TP53 pathway activation and reduce deleterious cellular stress responses in SDS (33).

When we analyzed the different Eif6 dosages in the *sbds* KO zebrafish model, we determined that there is a required critical Eif6 dosage. Both excessive and insufficient levels of Eif6 are detrimental, indicating that a precise amount is crucial for the survival of *sbds* KO zebrafish. The optimal amount appears to be around 50%–60% of the WT Eif6 levels. Jaako et al. (26) showed that EIF6 binds to free cytoplasmic 60S subunits, including the pre-60S and mature post-termination 60S in a translationally inactive state. This mechanism allows EIF6 to undergo recycling, reattaching to the 60S subunit after the ribosome has completed translation. This process ensures efficient utilization of EIF6 during the ribosomal cycle, facilitating proper ribosome assembly and function in cellular protein synthesis.

Somatic genetic rescue has been reported in multiple congenital blood disorders (34). Stochastically occurring mutations confer a fitness advantage. We postulate that somatic genetic rescue involving EIF6 in SDS confers an adaptive response by relieving the stress of stoichiometric imbalance (35) and reducing the level of EIF6 accumulation in the cytoplasm. This leads to a lessened magnitude of TP53 activation. In SDS, the loss of SBDS function perturbs ribosome biogenesis, leading to an accumulation of EIF6 in the cytoplasm. This imbalance produces cellular stress and triggers TP53 activation. However, somatic genetic rescue conferred by reduced EIF6 levels leads to its decreased cytosolic accumulation and counteracts this stress response by restoring a more balanced ribosomal protein stoichiometry. A lower magnitude of TP53 activation could promote cellular survival by modulating the time of appearance, levels of, and duration of TP53 (36). This adaptive response may be a critical factor in cellular survival and function in SDS, potentially explaining the variability in disease such as the fluctuations in hematologic counts or decreased requirements for pancreatic enzyme replacement (37–39). In other subclones within the same individual, stochastic loss of one *TP53* allele alleviates stress, reduces the likelihood of *EIF6* mutation (or deletion of 20q). This double-edge effect increases the likelihood of mutating the second *TP53* allele, which promotes the transformation to MDS/AML (40). On the other hand, reduced EIF6 protein levels provide a stress release, reducing the likelihood of TP53 in that cell and its progeny. Clones harboring mutations in EIF6 may reduce the risk of their own leukemic transformation, while other clones harboring TP53 mutations can transform to leukemia. Our animal model demonstrates that germline knockdown of *eif6* confers a modest benefit in survival. Future studies will determine at what level of Eif6 protein expression, as mitigated by CRISPR or missense mutation, can confer the maximal benefit in survival. Determining the dose of EIF6 reduction could guide the pharmacologic design to effect clinical somatic genetic rescue. Additional studies will characterize the clonal competition between subclones carrying *TP53* or *EIF6* mutations, although there may be additional environmental factors or exposures (such as infection).

This investigation provides insights into the relationship between ribosome biogenesis factors Sbds and Eif6, cellular stress responses mediated by the Tp53 pathway, the implications for developmental processes and pathogenesis, and a mechanism for somatic genetic rescue in SDS. Our zebrafish strains provide vertebrate models to test Eif6’s effects on translational efficiency through cytoplasmic accumulation and reduced ribosomal recycling (26). Understanding the regulatory networks involving EIF6 and TP53 provides valuable insights into the pathogenesis of these disorders and phenotypic variability and offers promising avenues for therapeutic intervention. Furthermore, there are TP53-independent pathways that contribute to the pathogenesis of SDS. We are currently identifying them.

## Methods

### Sex as a biological variable.

Both male and female participants with SDS provided material. Zebrafish were used prior to sexual development, and their sex was not considered a variable.

### Zebrafish husbandry and mutagenesis.

WT AB, *tp53^M214K^* (41) zebrafish were obtained from the Zebrafish International Research Center, ZIRC (https://zebrafish.org/). The *sbds^nu132^* (denoted as *sbds^-^*) was created in Northwestern University (4).All lines were maintained according to standard protocols (42). sgRNA targeting *eif6* exon 2 were designed with the Integrated DNA Technologies (IDT) design tool (Supplemental Table 1). sgRNA was prepared following the manufacturer’s protocol. Genomic DNA was extracted from embryos 1–2 days after injection for restriction site polymorphism-based genotyping. (Primer sequences are listed in Supplemental Table 1). All obtained alleles were sequenced from PCR products.

### Patient-derived tissues.

Patients with SDS were recruited during their annual visits at the Cystic Fibrosis Center in Verona, Italy. Patients with SDS were included only if they carried biallelic *SBDS* mutations without signs of MDS or AML, as indicated in Supplemental Table 2. During the outpatient scheduled visit, 1 additional peripheral blood sample (10 mL) and 1 additional bone marrow aspirate (5 mL) were collected in EDTA-containing tubes. BM-MNCs and PBMCs were isolated by Ficoll-Paque Plus (MilliporeSigma) density-gradient centrifugation at 400*g* for 25 minutes at room temperature. To generate LCLs from patients with SDS and healthy donors, peripheral B cells (CD45^+^CD19^+^) were isolated with the Rosette Sep B Lymphocyte Kit (Miltenyi Biotec). B cells were infected for 18 hours with EBV isolated from marmoset blood leukocyte B95.8 virus-producing cells. Once generated, LCLs with a comparable number of cell culture passages were incubated for 24 hours in RPMI-1640 medium (MilliporeSigma), supplemented with 10% FBS (MilliporeSigma).

### RT-qPCR.

At least 3 biological replicates were used in each experiment, with all 3 genotypes from the same clutch. Pools of 7–10 larvae were homogenized, and RNA was extracted using TRIzol (Invitrogen, Thermo Fisher Scientific). cDNA was synthesized with the iScript cDNA Synthesis Kit (Bio-Rad). Human LCLs were cultured in complete RPMI-1640 with 10% FBS. Cells were lysed, and total mRNA was extracted using the High Pure mRNA Extraction Kit (Roche). cDNA was synthesized using the High-Capacity cDNA Reverse Transcription Kit (Thermo Fisher Scientific). For RT-qPCR, cDNA was mixed with PowerUP SYBR Green Mix (Thermo Fisher Scientific). mRNA expression in mutant fish and patients’ cells was normalized to β-actin and calculated using the ΔΔCt method. Primers are listed in Supplemental Table 3.

### Transient silencing of EIF6 in LCLs.

Transient silencing was performed using the TriFECTa RNAi Kit (hs.Ri.EIF6.13, IDT) according to the manufacturer’s recommendations. LCL cells were transfected with specific double-stranded siRNA molecules against *EIF6* (Supplemental Table 3) or with a negative control double-stranded RNA, complexed with Lipofectamine RNAiMax (Invitrogen, Thermo Fisher Scientific) cationic liposomes in serum-free RPMI-1640 for 12 hours. Then, cells were cultured for a further 12 hours in complete RPMI-1640 with 10% FBS. Gene silencing of *EIF6* was verified by both RT-qPCR and Western blotting, as mentioned above.

### RNA-Seq.

RNA was extracted from pools of 8–9 individually genotyped larvae at 10 dpf using TRIzol (Thermo Fisher Scientific). Three pools of *eif6^–/–^* or WT zebrafish cells from the same clutch were compared. RNA quality was determined with the Bioanalyzer (Agilent Technologies), and *eif6* mRNA expression was measured by RT-qPCR. RNA-Seq library preparation and sequencing and mapping of 3 pools of *eif6^–/–^* and 3 pools of *eif6^+/+^* were performed by the Beijing Genome Institute (4). Based on the fragments per kilobase of transcript per million mapped reads (FPKM) values (43), we used the EBSeq R package for differentially expressed gene detection between *eif6^–/–^* and *eif6^+/+^* (fold change >2, and *P <* 0.05). We performed gene enrichment analysis using WebGestalt (44) and searching for KEGG pathways. Heatmaps were made using Heatmapperclustering (http://heatmapper.ca/) (45).

### Western blot analysis.

After genotyping by fin-clipping the larvae, the rest of the body was kept in individual tubes and boiled for 3 minutes in Laemmli buffer (Bio-Rad). For human tissue, total proteins were extracted in RIPA buffer supplemented with complete protease inhibitor EDTA-free cocktail (Roche). The antibodies used are listed in Supplemental Table 4.

### Detection of neutrophils.

Detection of neutrophils was performed as previously described (17). *o-*Dianisidine staining was performed as described previously (46) and correlated with the genotype.

### Polysome profile.

Pools of 10–25 larvae (5 dpf) from *eif6^+/+^*, *eif6^del2aa/del2aa^*, *eif6^ms/ms^*, and *eif6^–/–^* larvae were collected and treated with cycloheximide 100 μg/mL for 10 minutes and then sacrificed and stored at –80°C. Larvae were thawed and lysed at 4°C in polysome lysis buffer according to a previously described protocol (4). Polysome profiles were done in triplicate for the 4 genotypes.

### Statistics.

Descriptive and analytical statistics were performed using GraphPad Prism 6.0 (GraphPad Software). Parametric data are shown as the mean ± SEM, with *n* values represented by dots in histograms, each indicating a different animal. Statistical tests included unpaired, 2-tailed *t* tests or 1-way ANOVA with Tukey’s or Dunnett’s multiple-comparison test. A *P* value of less than 0.05 was considered significant.

### Study approval.

Zebrafish experiments were approved by the IACUC of the Cleveland Clinic. Human samples were obtained in compliance with the Declaration of Helsinki, after written consent, with protocols approved by the ethics committee of Azienda Ospedaliera Universitaria Integrata (protocol 4182CESC; approval no. 423, 04/06/2023).

### Data availability.

RNA-Seq data are available in the Gene Expression Omnibus (GEO) database (GEO GSE282310). All other data are available upon reasonable request to the corresponding authors.

## Author contributions

UO and VB designed studies, performed experiments, acquired and analyzed data, and wrote the manuscript. MS, CB, and AS performed experiments. EC analyzed data and wrote the manuscript. MC provided reagents and wrote the manuscript. SJC designed studies, analyzed data, and wrote the manuscript.

## Supplementary Material

Supplemental data

Unedited blot and gel images

Supporting data values

## Figures and Tables

**Table 1:**
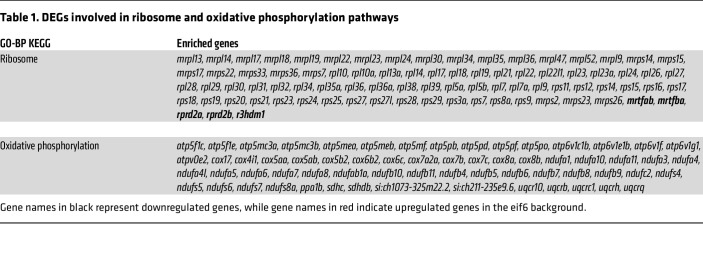
DEGs involved in ribosome and oxidative phosphorylation pathways

## Version 1 02/18/2025

In-Press Preview

## Version 2 04/15/2025

Electronic publication
