# Supplementary material for: Reduced EIF6 dosage attenuates TP53 activation in models of Shwachman-Diamond syndrome
Source: J Clin Invest. 2025 Feb 18;135(8):e187778. doi: 10.1172/JCI187778 (PMC11996912; doi:10.1172/JCI187778)

Supplemental Tables

Supplemental Table 1. Zebrafish *eif6* gRNA sequence and primers

eif6 gRNA sequence			
**Gene**	**CRISPR target sequence (5' - 3')**	**RE site lost**	
eif6	ACATACTGTTTGGTAGCAAT	MfeI	
**Primers for zebrafish genotyping**			
**Gene**	**F Primer (5' - 3')**	**R Primer (5' - 3')**	**RE**
sbds	TGATGAAGTCCTGCAAACCA	CAGGTCATCTGTTCCAAAAGC	
eif6	CAGTTCGTGCATCATTTGAAAAG	CTGTAAAAATTTTCTGAACC	MfeI, RsaI
tp53	ACATGAAATTGCCAGAGTATGTGT	TCGGATAGCCTAGTGCGAGC	MboII
**Zebrafish RT-qPCR primers**			
**Gene**	**F Primer (5' - 3')**	**R Primer (5' - 3')**	
atf4b	GGTGTCTCTGCCCTGGTTAC	TCGTTCTGTCATCCAATCCA	
atf6	CTGTGGTGAAACCTCCACCT	CATGGTGACCACAGGAGATG	
bax	GGAGATGAGCTGGATGGAAA	AGATCTCACGGGCCACTCT	
bip	AGATCTTCTCCACTGCTTCCGACAA	TCTACAGCTCGTCCTTCTCTTCGGC	
casp9	CTGTCAAAGGGGGTCTTCAC	TCGTCTCCAGGTCTTTCACC	
ccng1	CTTCTGTGCGGAGACGTTTT	ACAGCGATGTAGAAGCAGCA	
cdkn1a	AGAGCTCGCGTGGAGTCAG	CCGAAAAGACTCCGCCTA	
cdkn2ab	CGGAAACATTTCCCATGTTC	AAGGTGCGTTACCCATCATC	
chop	AAGGAAAGTGCAGGAGCTGA	TCACGCTCTCCACAAGAAGA	
eif6	CCCAACAACACGACAGATCA	TCGTTACAAGCGATGACGTT	
puma	ACT GCC CCA CAT CCC CTC AC	CGT CCC CGA TTG TCC TCA GTT G	
sbds	CCAACACAGTCTTTGTGAATG	CGCTTGGTCTCAGGATTC	
tp53	CTCTCCCACCAACATCCACT	ACGTCCACCACCATTTGAAC	
xbp-s	TGTTGCGAGACAAGACGA	CCTGCACCTGCTGCGGACT	
xbps-t	GAGGAGCCCACAAAGTCCTC	CGAAGTGCTTTTTCCTCTGG	
β-actin	TGCTGTTTTCCCCTCCATTG	TTCTGTCCCATGCCAACCA	

RE, Restriction Enzyme

Supplemental Table 2. Genetics and clinical aspects of SDS patients enrolled in this study. UPN, unique patient number; FTT, failure to thrive; PI, pancreas insufficiency; PS, pancreas sufficiency.

UPN	Sex	Age	Genotype	PMN (Cell/mm^3)	Phenotype	Cytogenetics
6	M	25	258+2T>C/101A>T	3520	PI, FTT, bone malformation, thrombocytopenia	46,XY del(20)q
13	M	25	258+2T>C/258+2T>C+183-184TA>CT	1480	PI, bone malformation, thrombocytopenia, anemia	46,XY del(20)q
24	F	29	258+2T>C/183-184TA>CT	500	PS, FTT, thrombocytopenia, anemia	46,XX
26	M	19	258+2T>C/183-184TA>CT	490	PI, FTT, bone malformation, thrombocytopenia	46,XY
37	F	10	258+2T>C/183-184TA>CT	250	PI, FTT, recurrent infections	46,XX i(7)(q10)
39	M	44	258+2T>C/183-184TA>CT	1390	PI, FTT, bone malformation, thrombocytopenia, cognitive impairment	46,XY
40	F	17	258+2T>C/183-184TA>CT	1040	PI, FTT, recurrent infections, HbF>2%, bone malformation, thrombocytopenia, anemia	46,XX i(7)(q10)
58	M	16	258+2T>C/183-184TA>CT	230	PI, FTT, HbF>2%, bone malformation, thrombocytopenia, anemia	46,XY
63	M	19	258+2T>C/258+2T>C+183-184TA>CT	1290	PI, FTT, bone malformation	46,XY
64	M	33	258+2T>C/624+1G>C	2460	PI, bone malformation, thrombocytopenia	46,XY
72	M	33	258+2T>C/183-184TA>CT	430	PI, FTT, recurrent infections, bone malformation, thrombocytopenia, cognitive impairment	46,XY
74	M	11	258+2T>C/183-184TA>CT	1320	PI, FTT, HbF>2%, cognitive impairment	46,XY
75	F	10	258+2T>C/183-184TA>CT	2280	PI, FTT, HbF>2%, bone malformation, thrombocytopenia, cognitive impairment	46,XX
82	M	16	258+2T>C/183-184TA>CT	300	PI, FTT, recurrent infections, bone malformation, thrombocytopenia, anemia, cognitive impairment	46,XY
87	M	18	258+2T>C/183-184TA>CT	1290	PI, FTT, recurrent infections, bone malformation, cognitive impairment	46,XY
150	M	7	258+2T>C/258+2T>C	1370	PI, FTT, bone malformation	46,XY
157	F	6	258+2T>C/258+533_459+403del	890	PI, FTT, bone malformation	46,XX

Supplemental Table 3. Human siRNA and RT-qPCR primers

**TriFECTa RNAi Kit (hs.Ri.EIF6.13)**		
**Gene**	**Name**	**Company**
EIF6	*hs.Ri.EIF6.13.1*	IDT
EIF6	*hs.Ri.EIF6.13.2*	IDT
EIF6	*hs.Ri.EIF6.13.3*	IDT
scrambled	DS NC1	IDT
**Human RT-qPCR primers**		
**Gene**	**Primer Name**	**Company**
SBDS	QT00018466	Qiagen
EIF6	QT00086933	Qiagen
TP53	QT00060235	Qiagen
CDKN1A	QT00062090	Qiagen
β-actin	QT01680476	Qiagen

Supplemental Table 4. List of antibodies used in Western blotting

Protein	Company	Dilution	Catalog number	Species used
SBDS	Santa Cruz	1:1000	SC-271600	Zebrafish
EIF6	NovusBio	1:500	NBP2-16975	Zebrafish
RPL11	Cell Signaling Technology	1:1000	CST-18163	Zebrafish
RPL5	Cell Signaling Technology	1:1000	CST-14568	Zebrafish
RPL26	Cell Signaling Technology	1:1000	CST-5400	Zebrafish
RPL22	Santa Cruz	1:1000	SC-373993	Zebrafish
RPL23	Abcam	1:1000	ab241087	Zebrafish
RPS3	Cell Signaling Technology	1:1000	CST-2579	Zebrafish
β-actin	Santa Cruz	1:3000	SC-47778	Zebrafish
SBDS	Abcam	1:300	ab128946	Human
EIF6	Abcam	1:1000	ab175383	Human
Tp53	Abcam	1:1000	ab131442	Human
p21	Abcam	1:1000	ab109199	Human
β-actin	Merck	1:5000	A3854	Human

Supplemental figure

Supplemental Figure 1. Eif6 mutants can produce neutrophils and erythrocytes. (A) Sudan black for neutrophil staining and (B) Staining of hemoglobin by O-dianisidine at 5 dpf. White star shows the swim bladder.

Figure S1

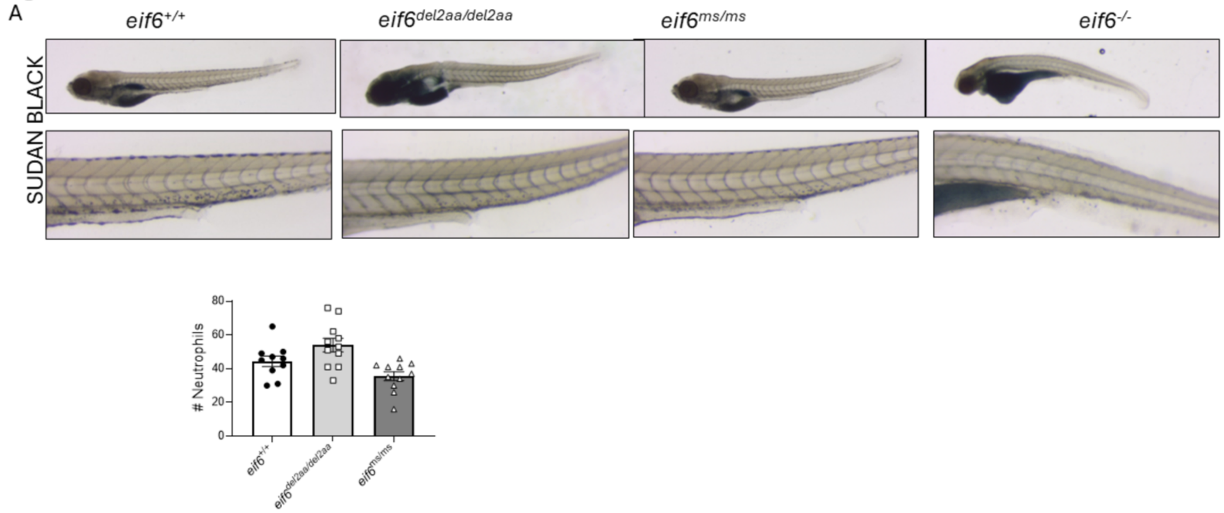

Supplemental Figure 1

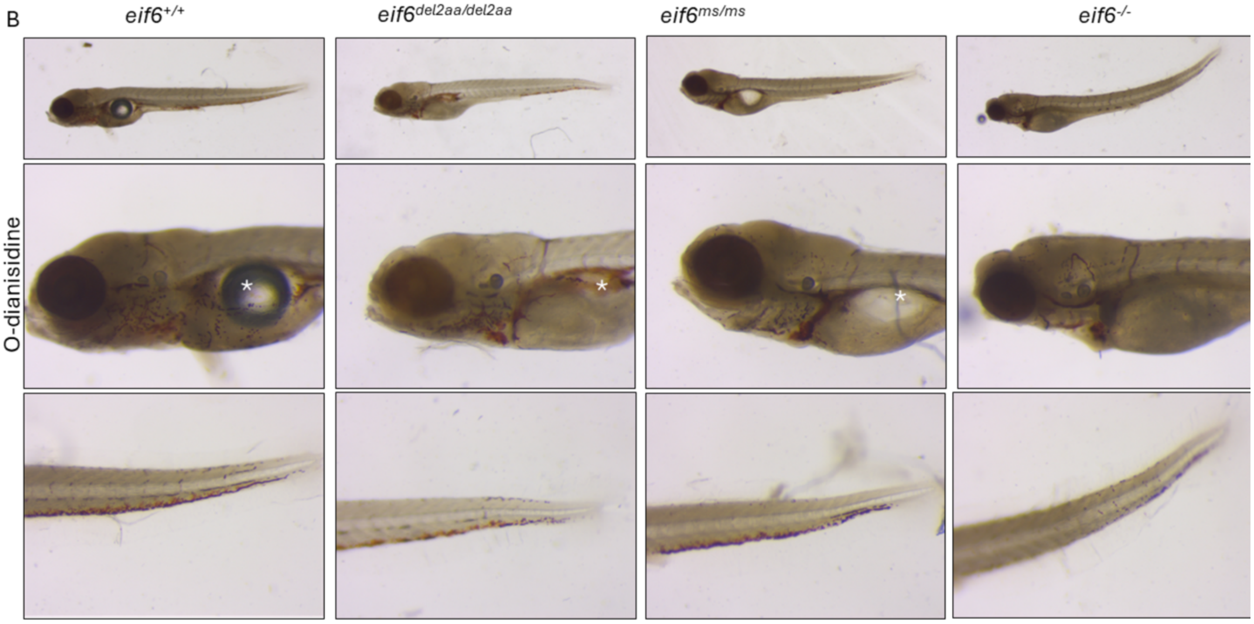

Supplement: Supplemental data [file jci-135-187778-s146.pdf]
