# Supplementary material for: Reduced EIF6 dosage attenuates TP53 activation in models of Shwachman-Diamond syndrome
Source: J Clin Invest. 2025 Feb 18;135(8):e187778. doi: 10.1172/JCI187778 (PMC11996912; doi:10.1172/JCI187778)

Figure 1A

EIF6

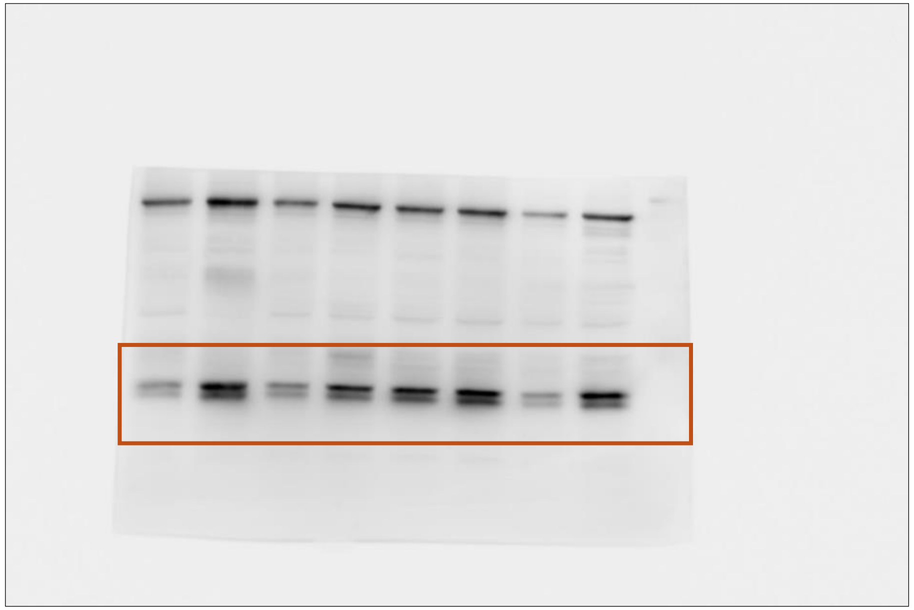

SBDS

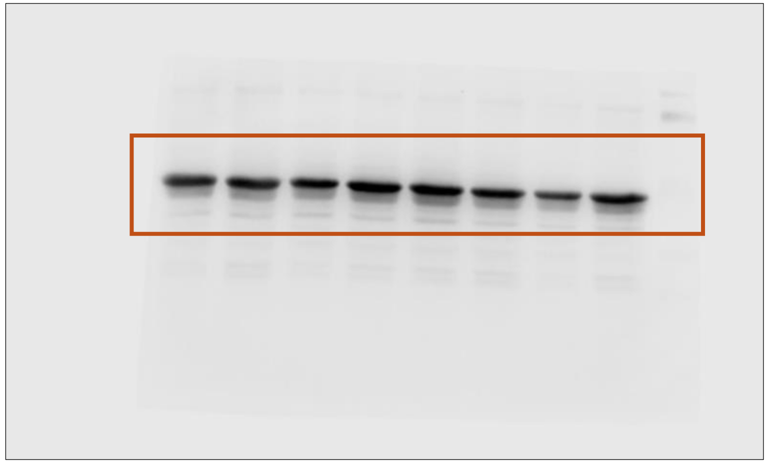

Figure 1B

SBDS

eIF6

β-Actin

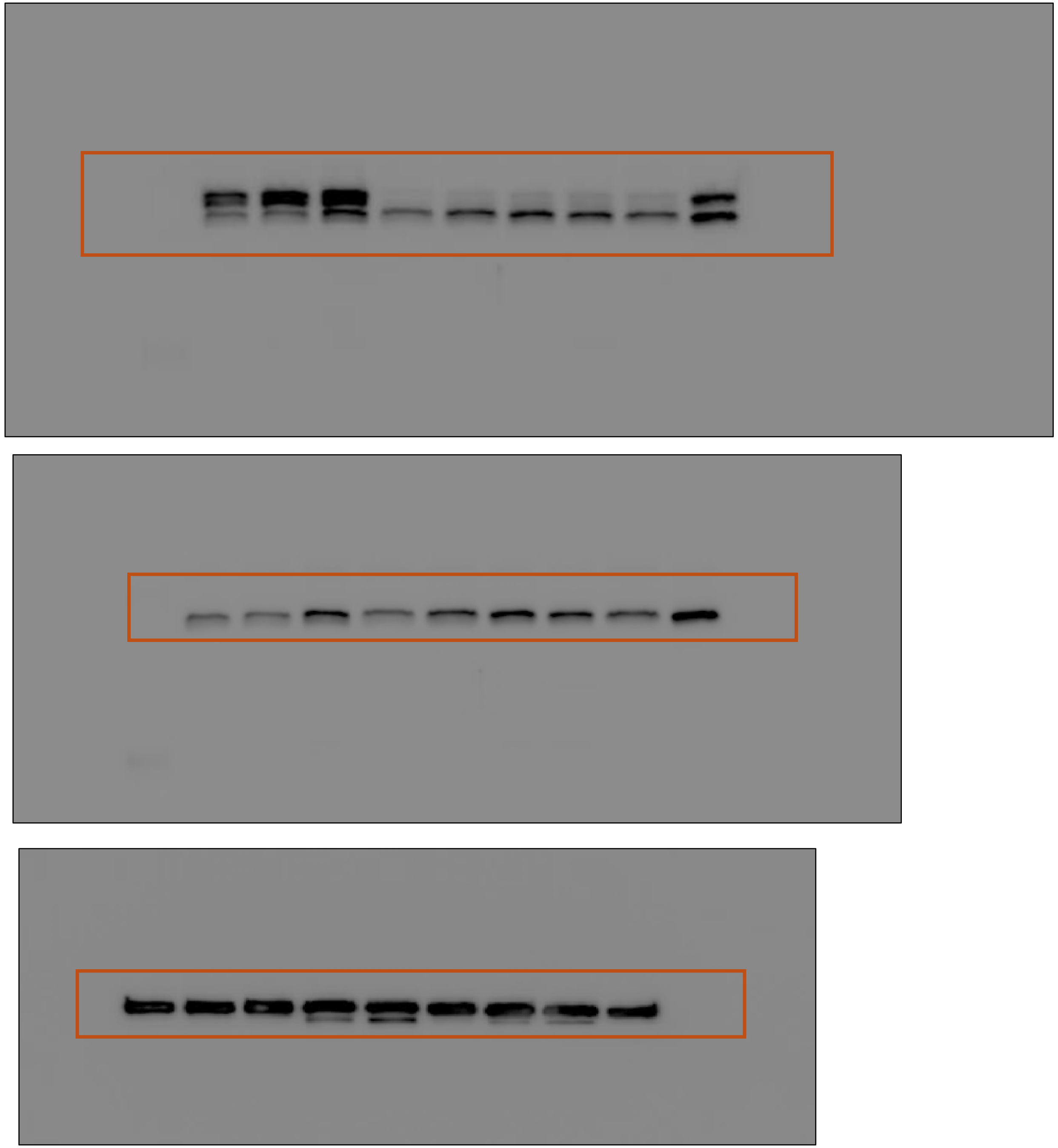

Figure 1C

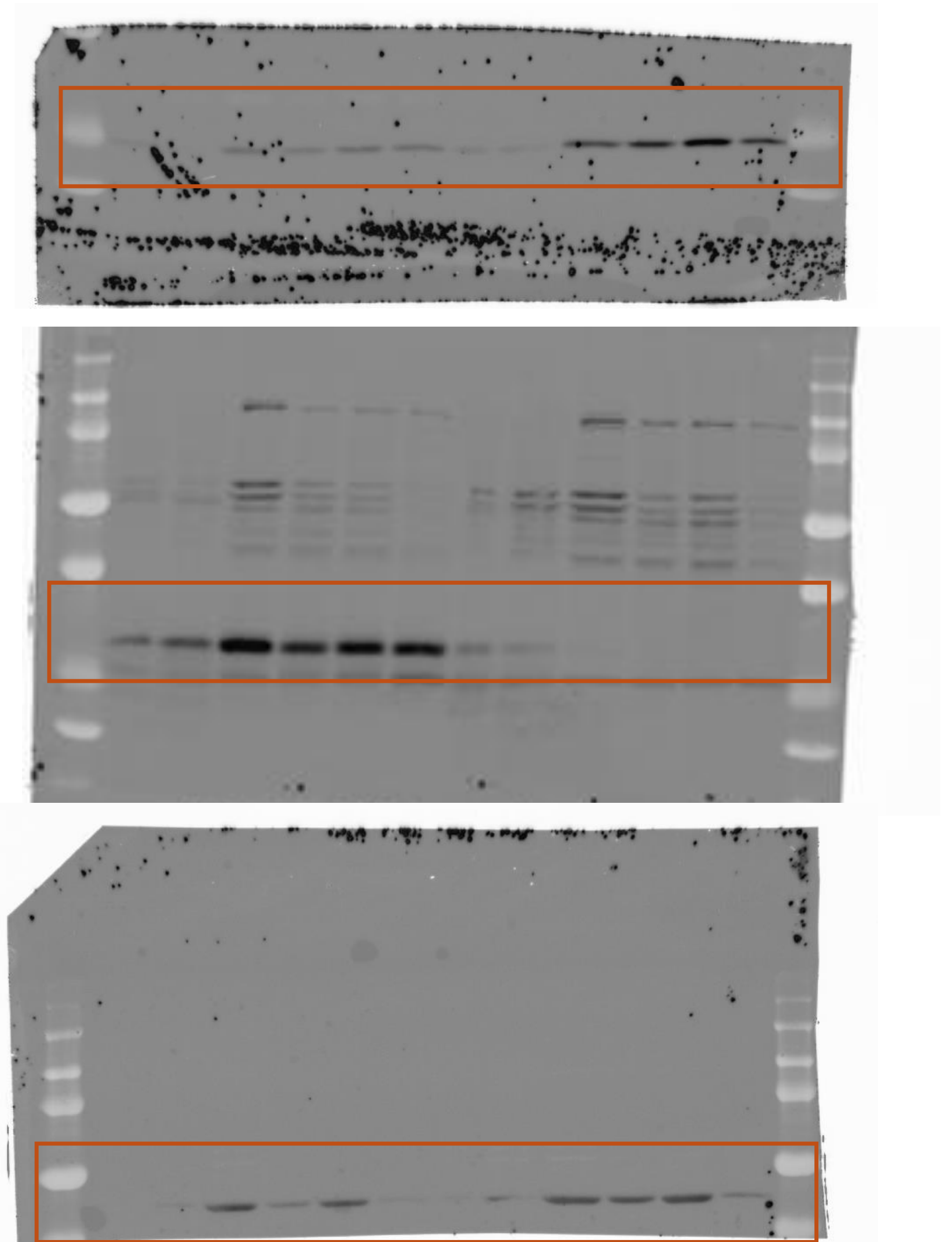

Figure 2C

Eif6

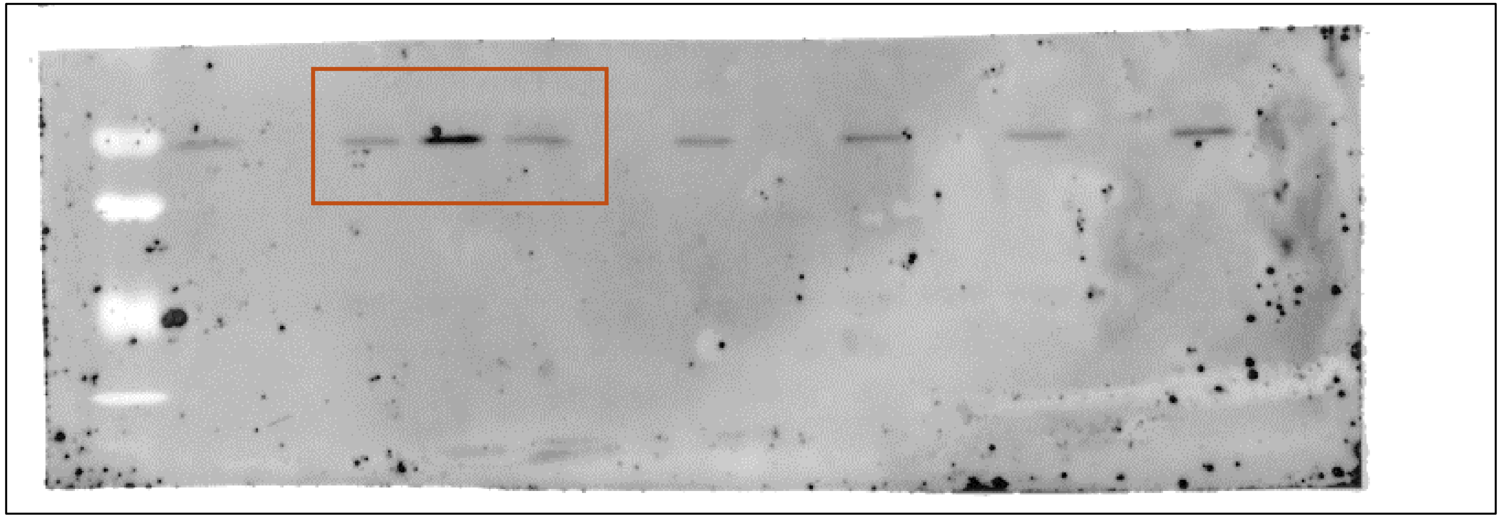

Act

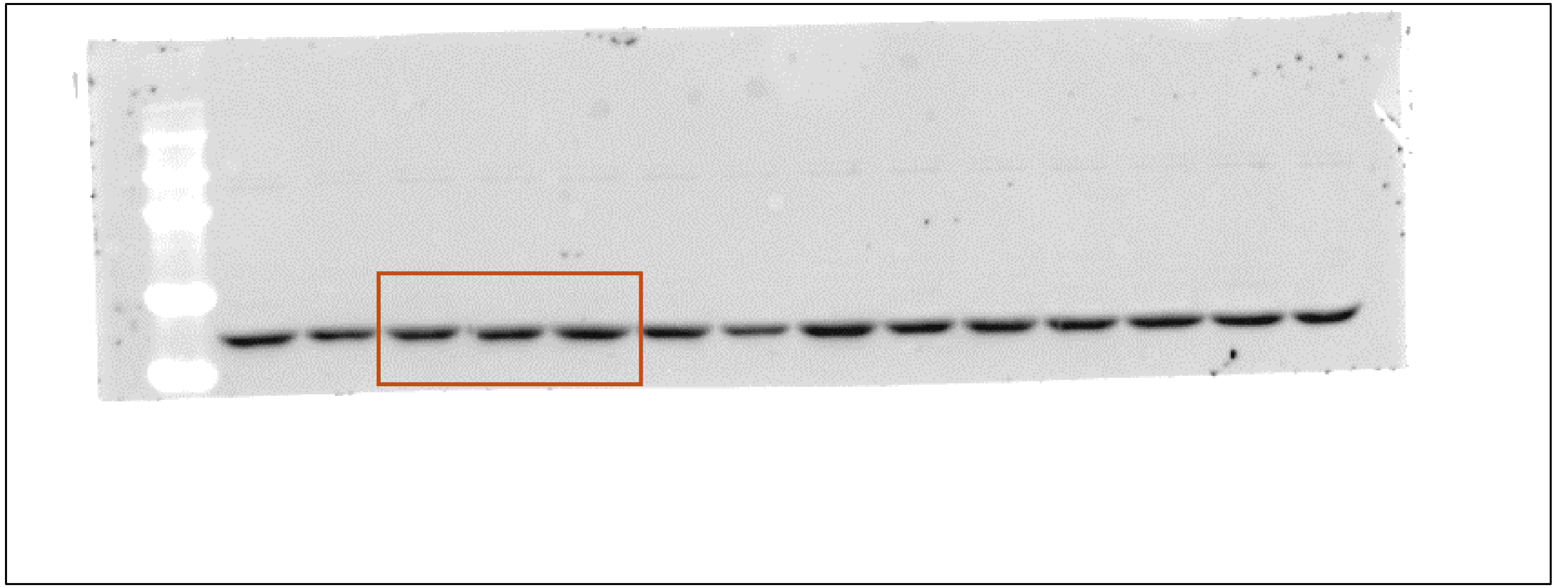

Figure 3C

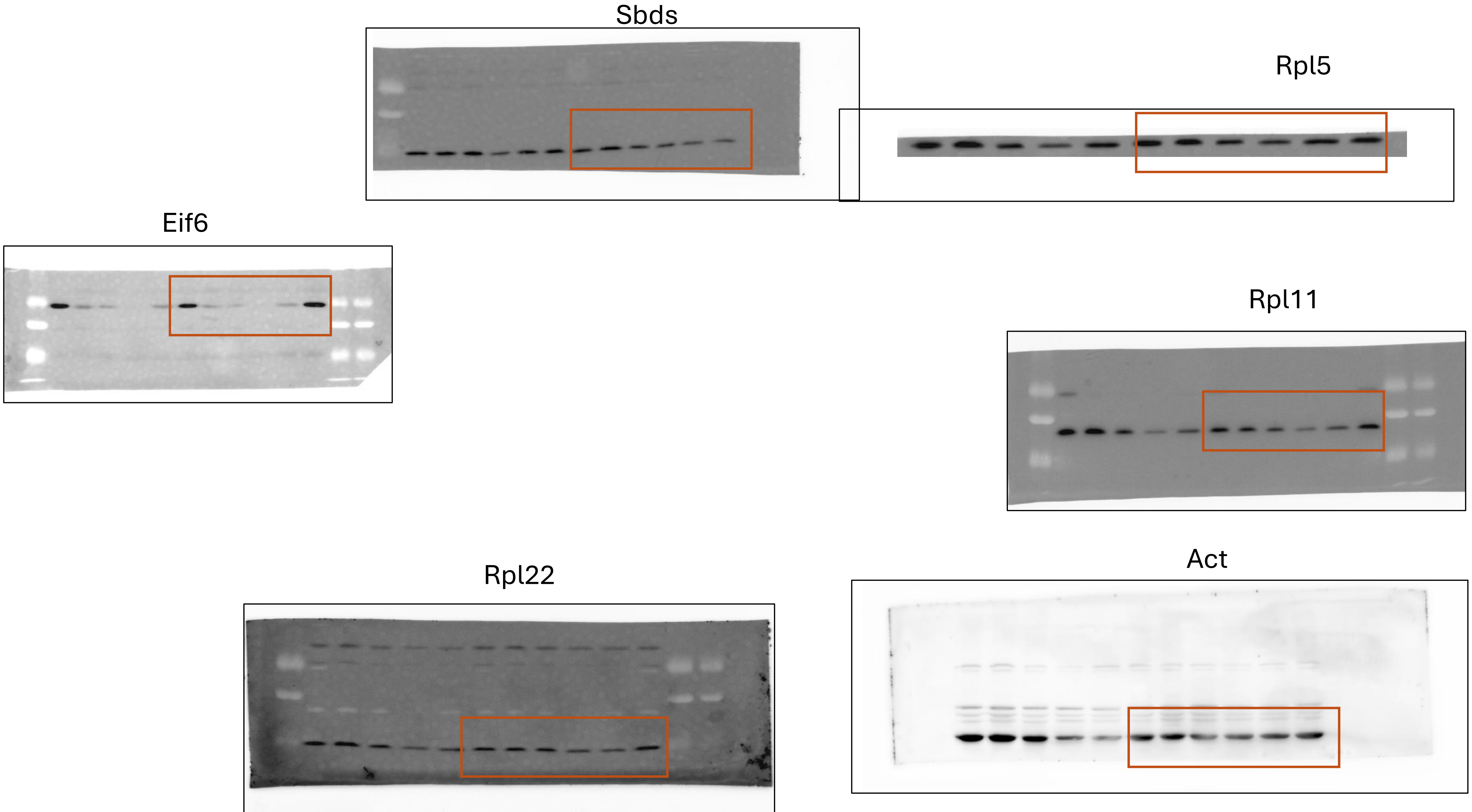

Figure 3E

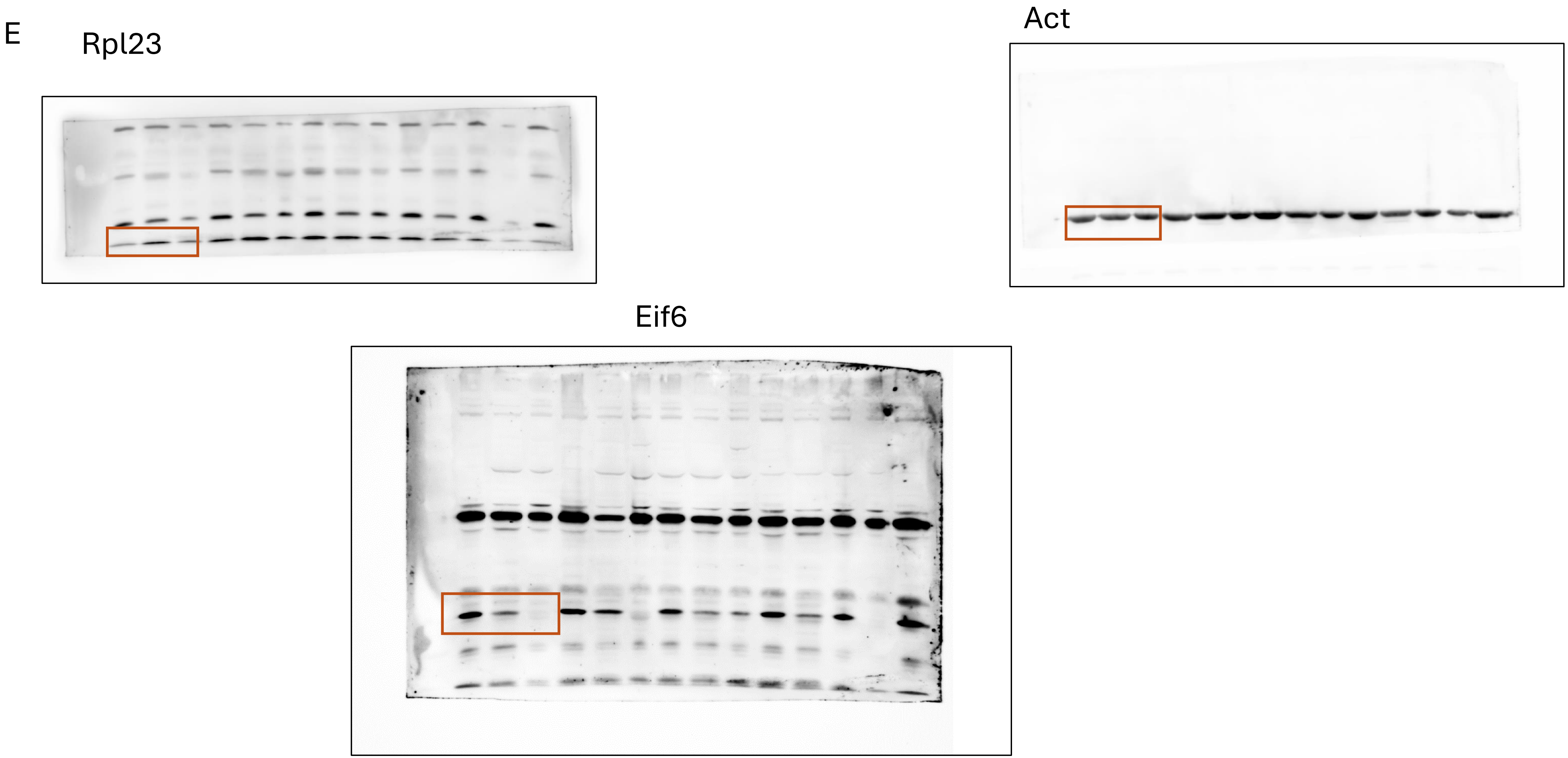

Figure 3E

Rps3

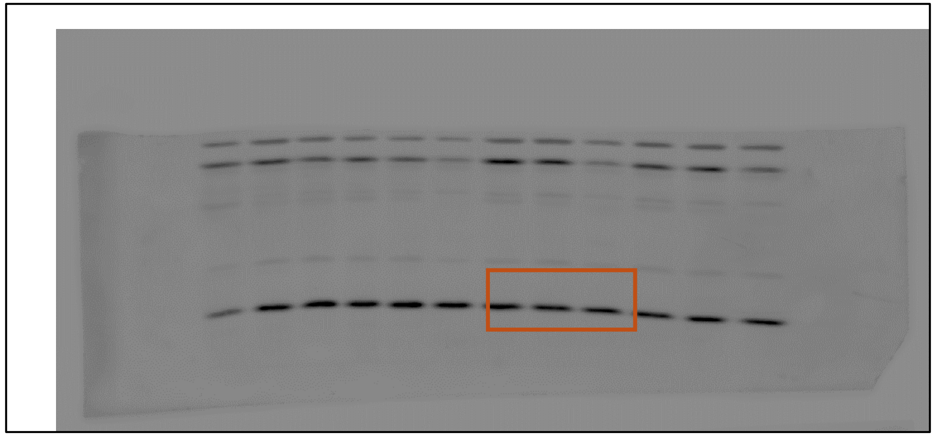

Eif6

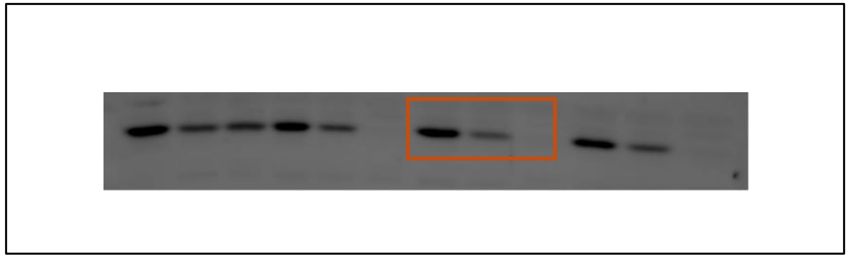

Act

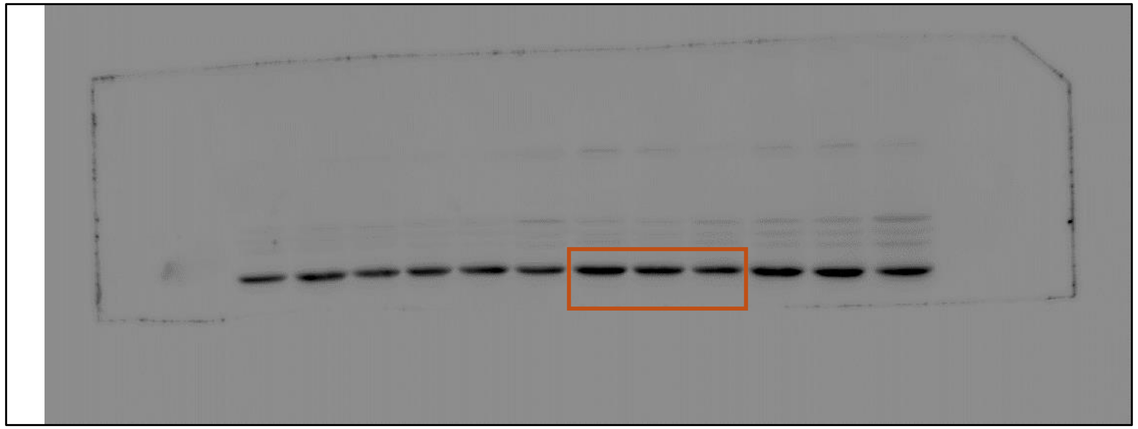

Figure 3G

Rps3

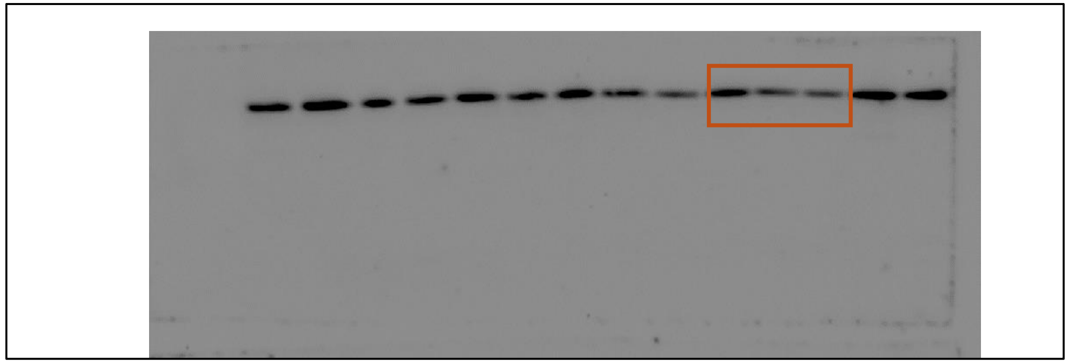

Act

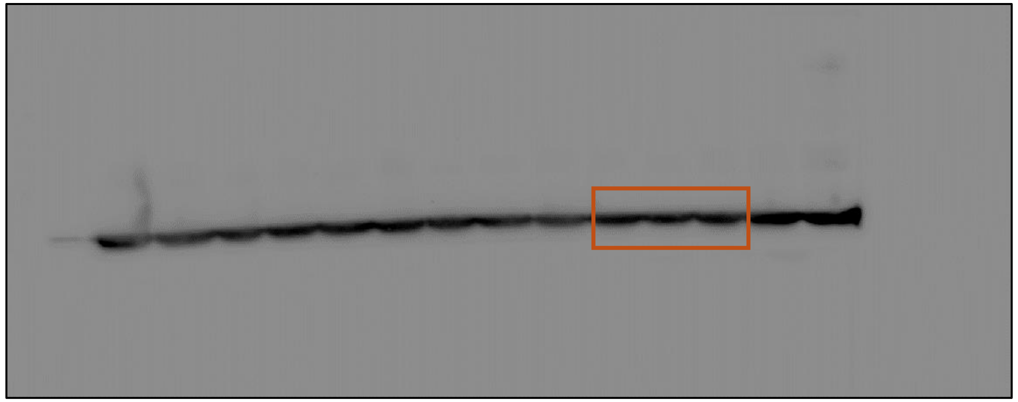

Eif6

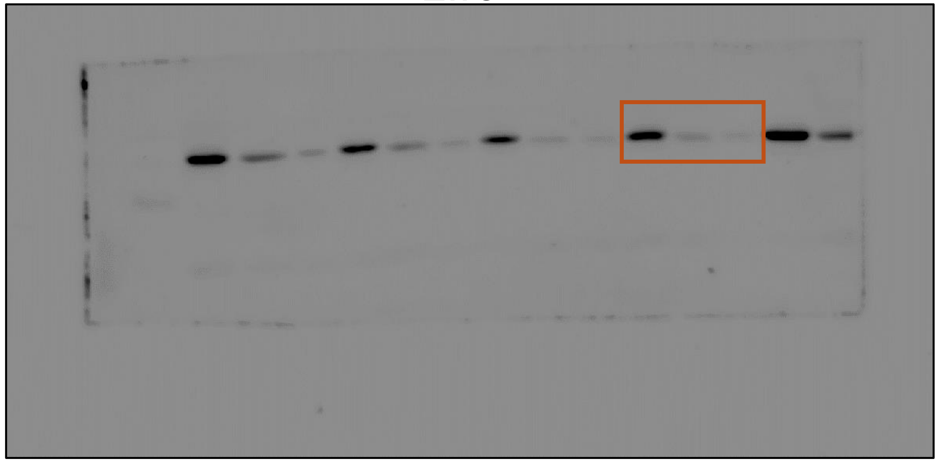

Figure 3G

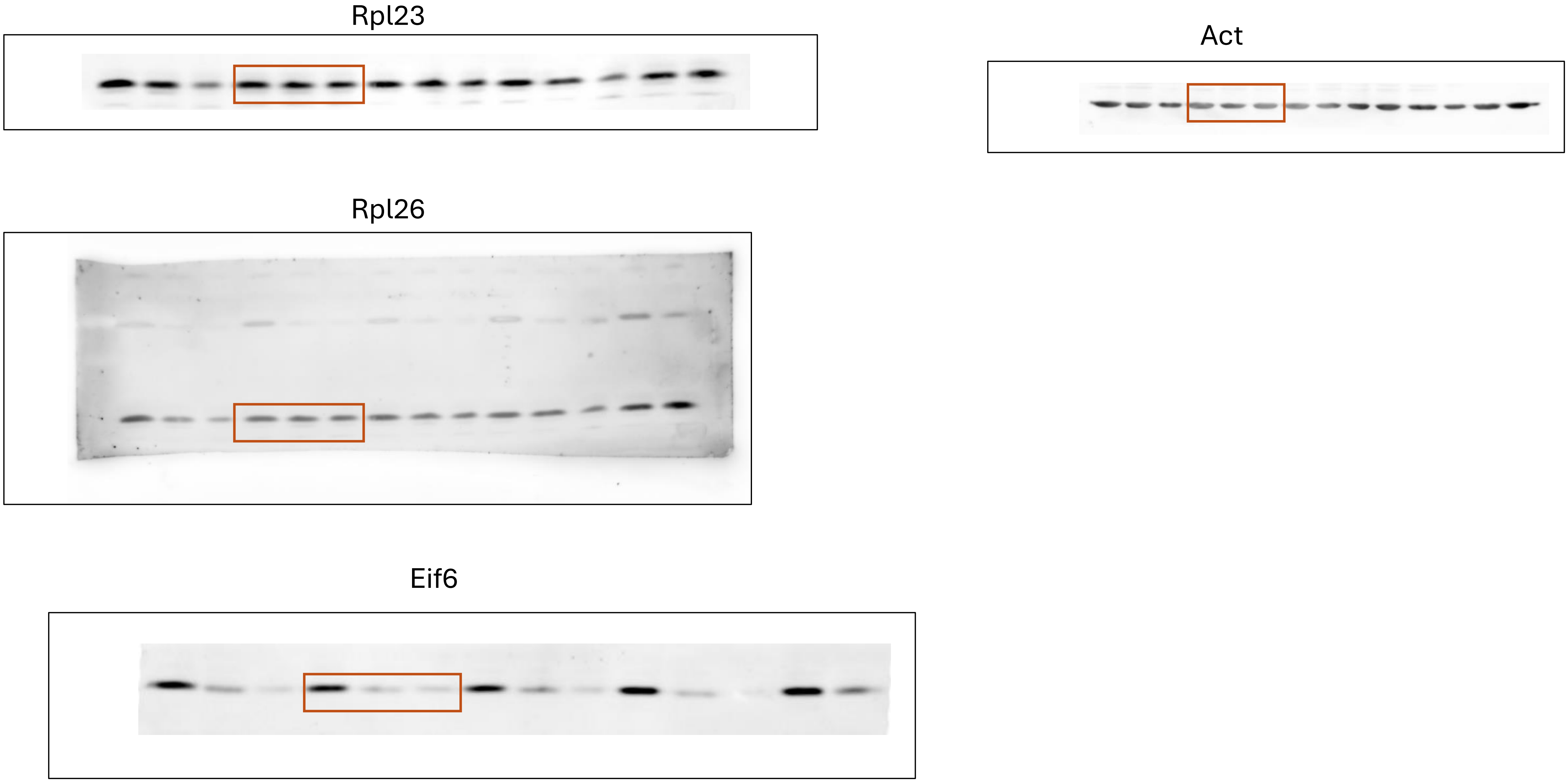

Figure 5C

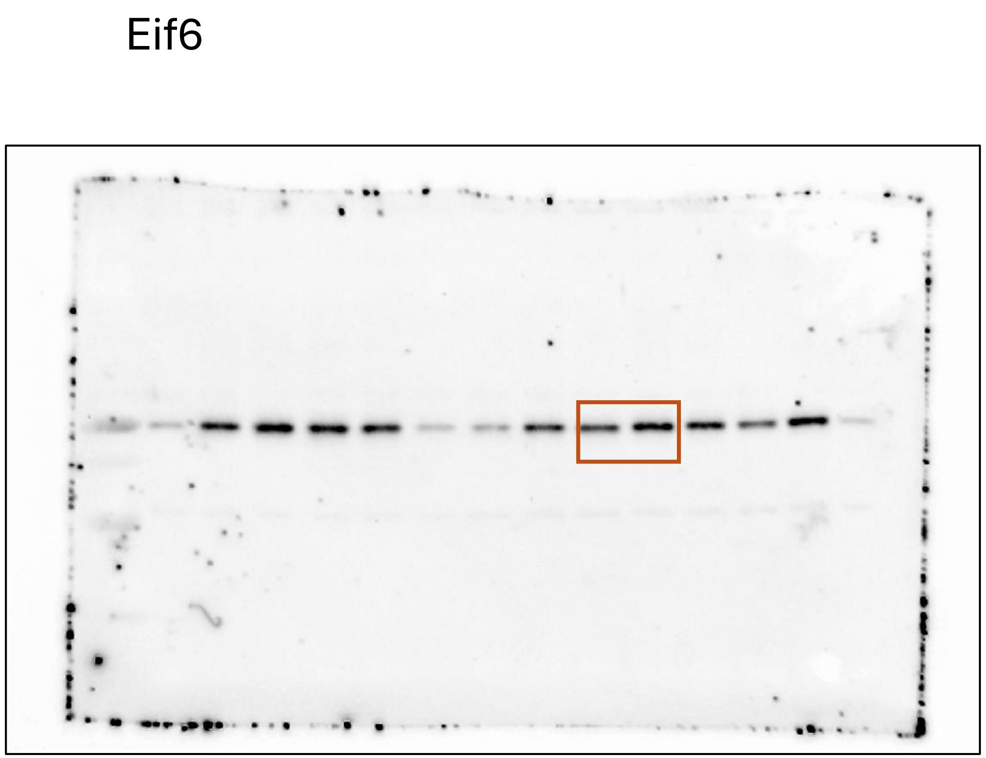

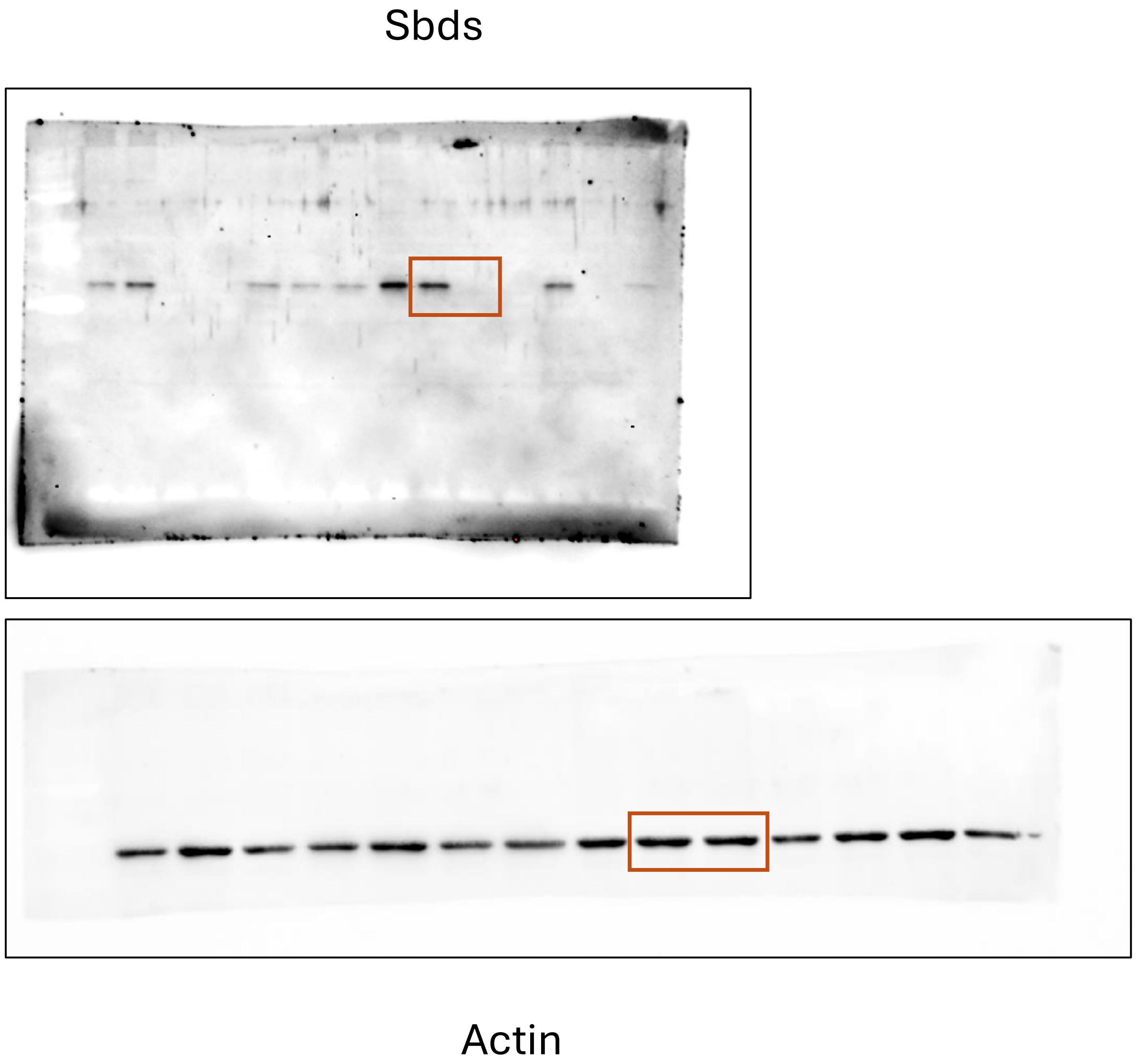

Figure 6C

Eif6

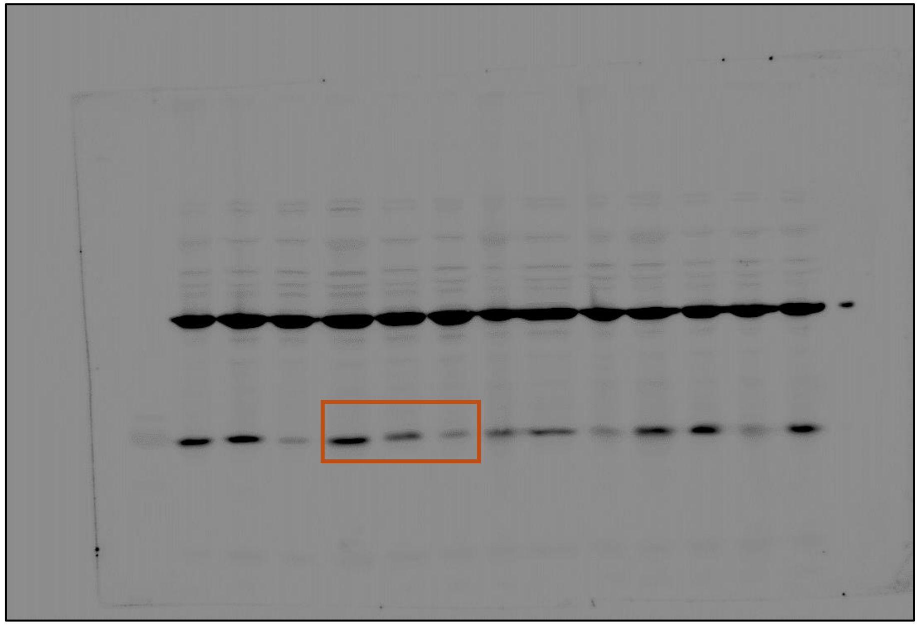

Act

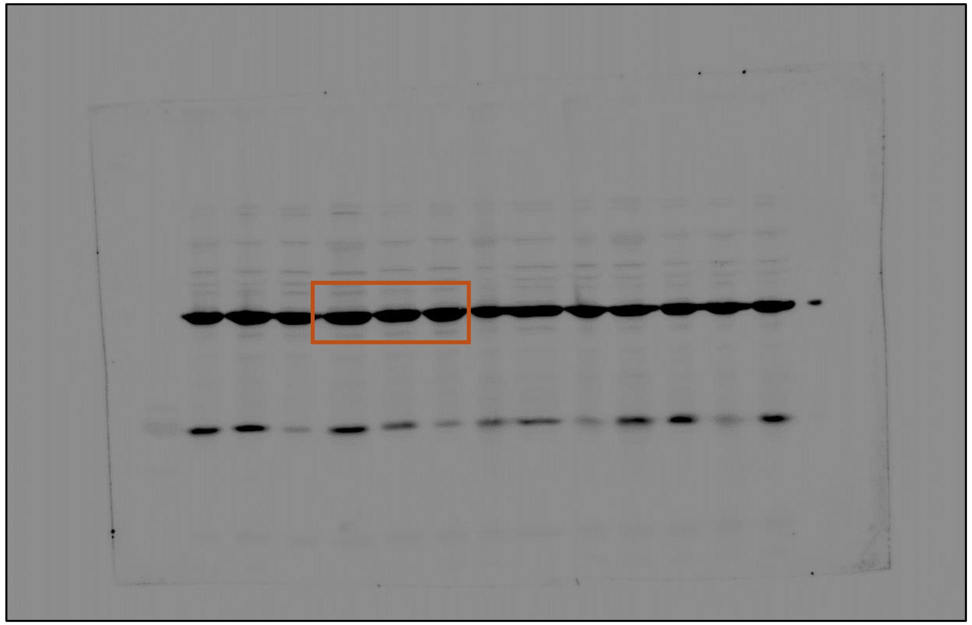

Figure 6E

Eif6

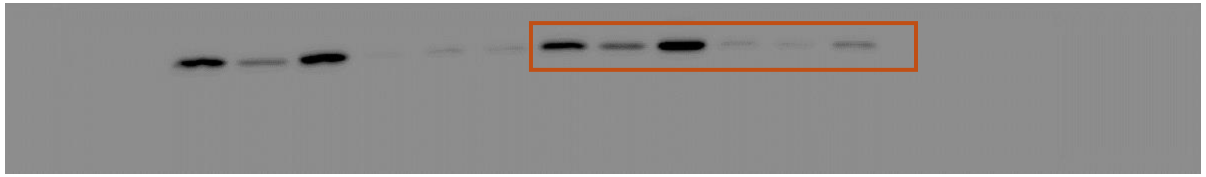

Act

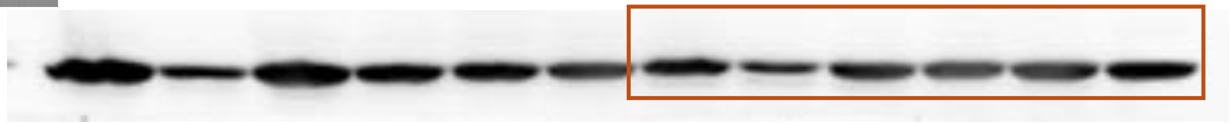

Sbds

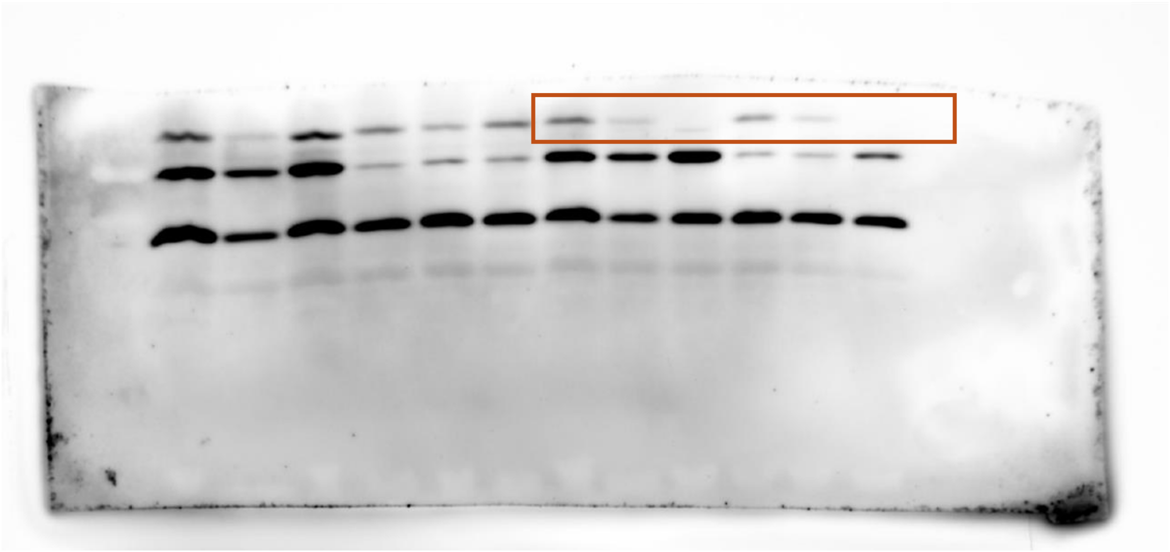

Figure 7A

p53

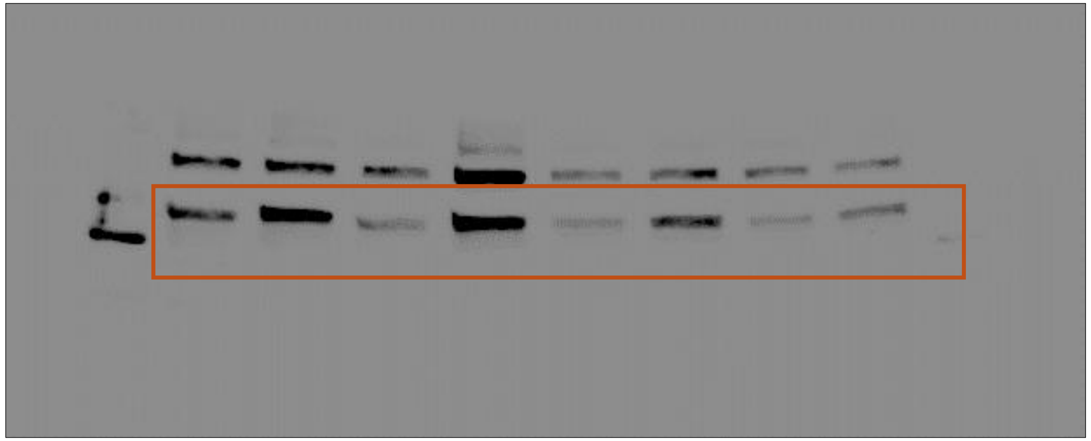

p21

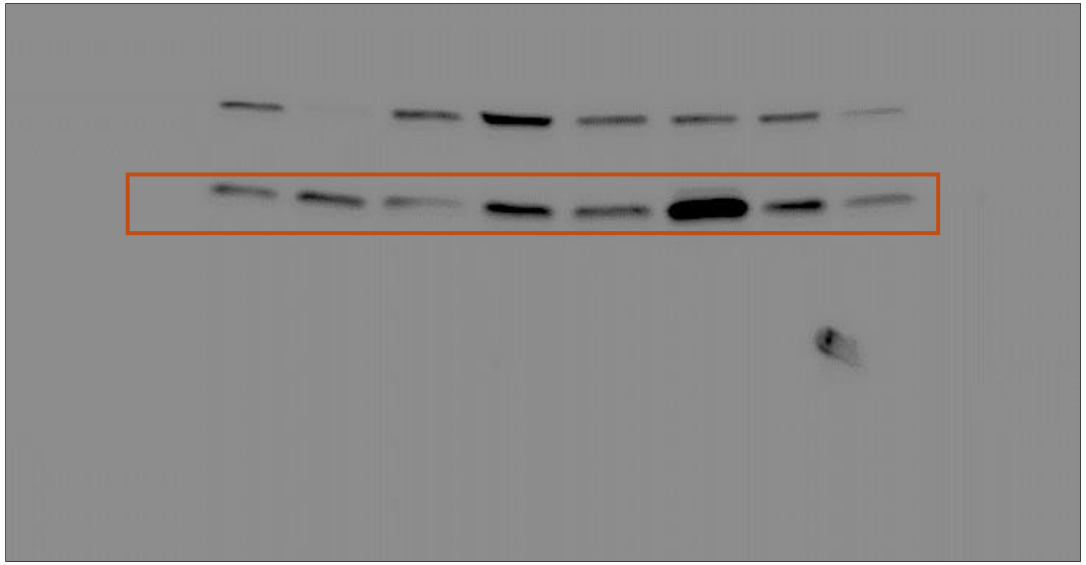

β-ACTIN

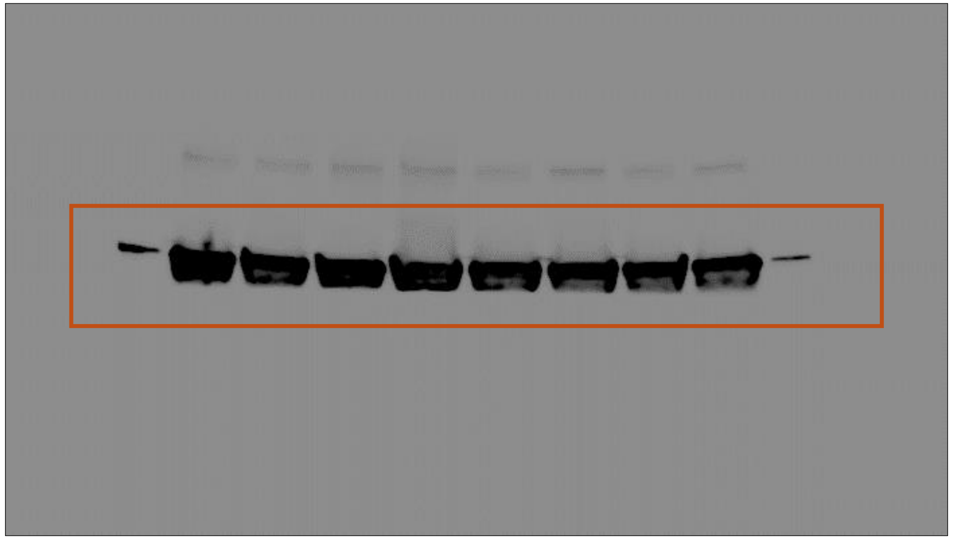

Figure 7D

eIF6

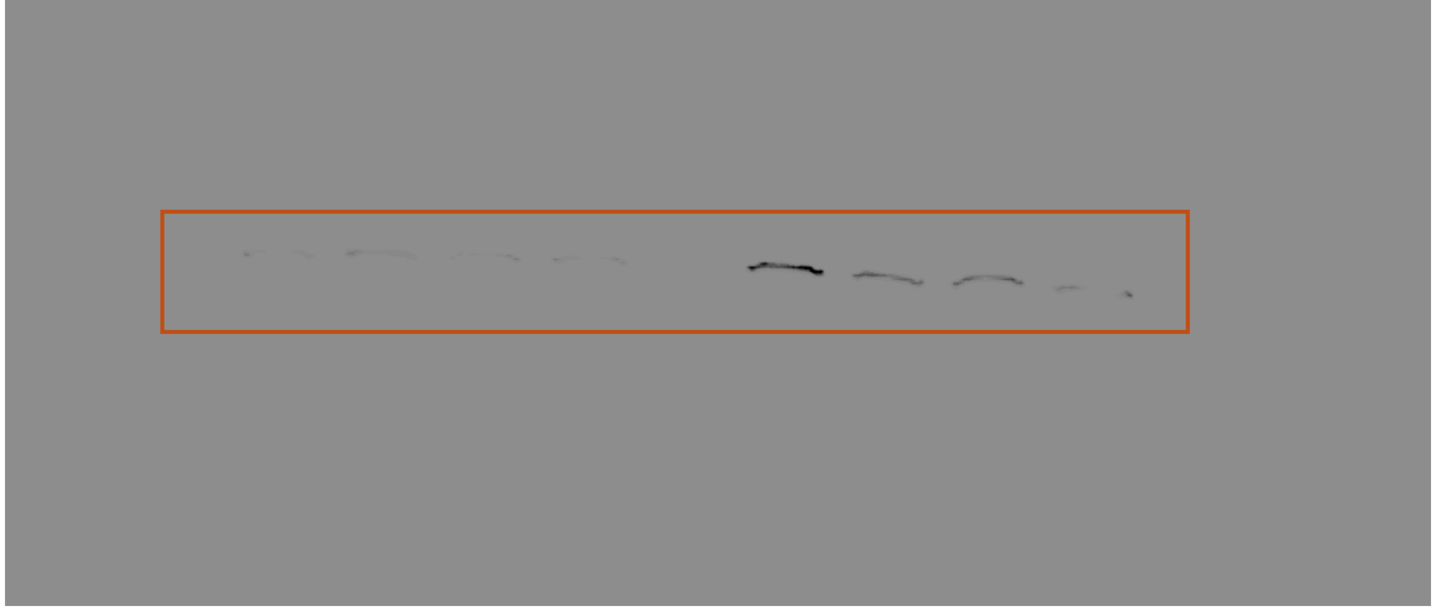

β-Actin

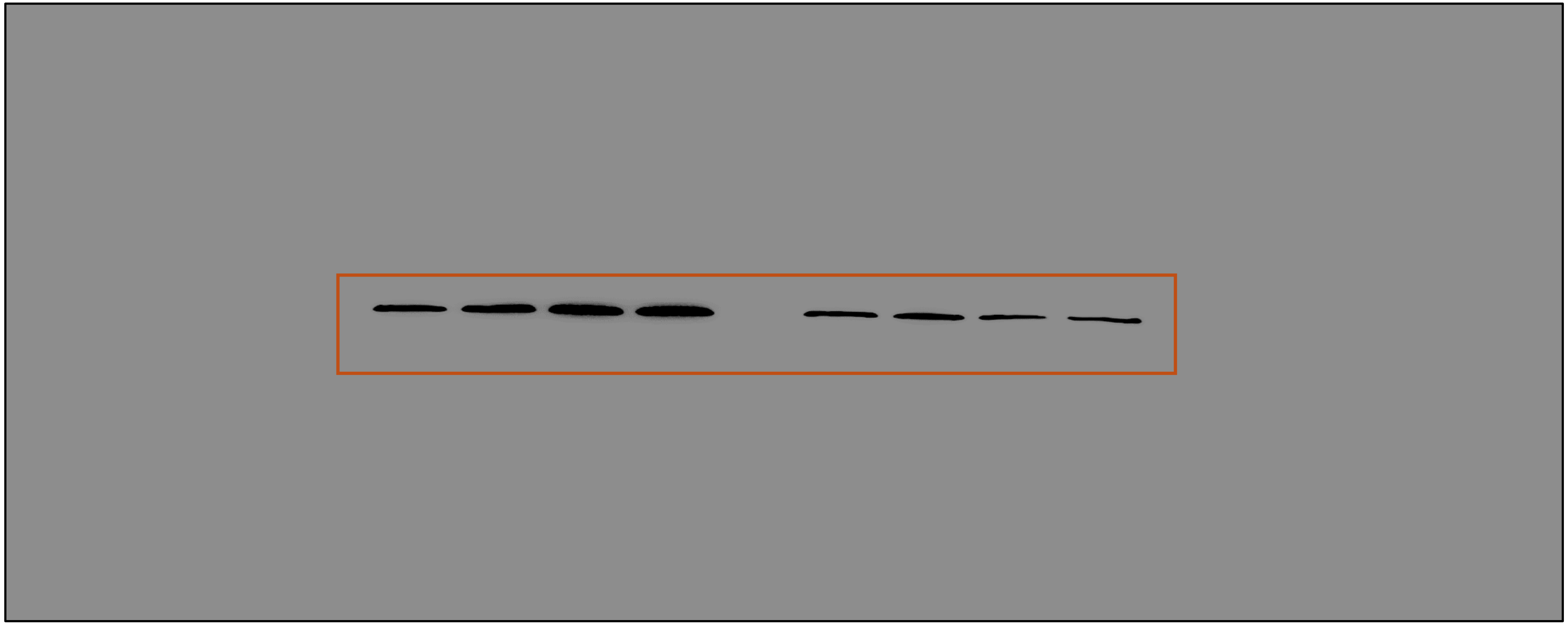

Figure 7G

p53

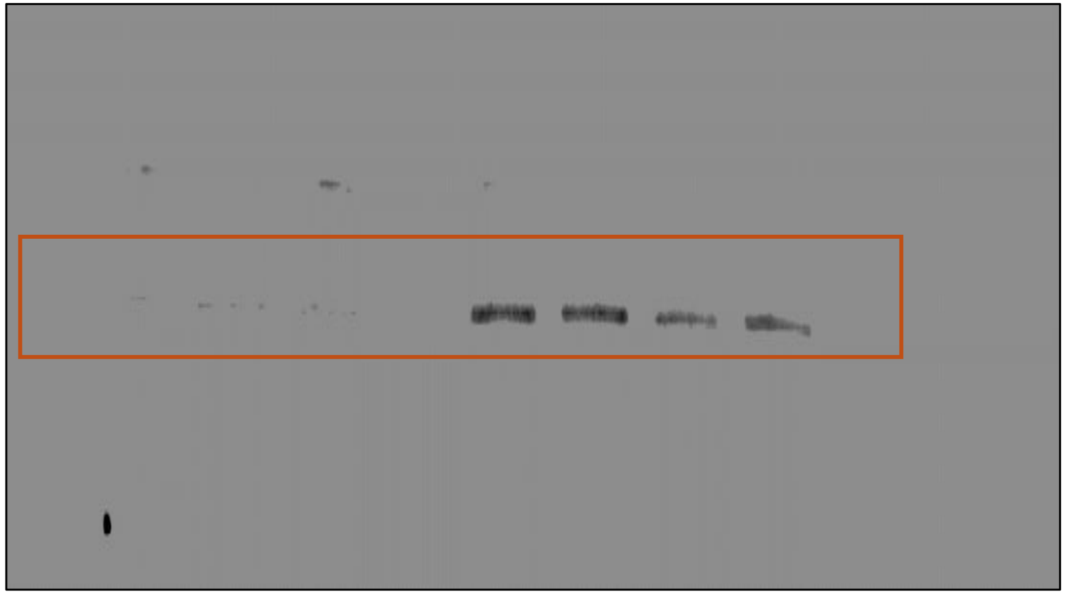

β-Actin

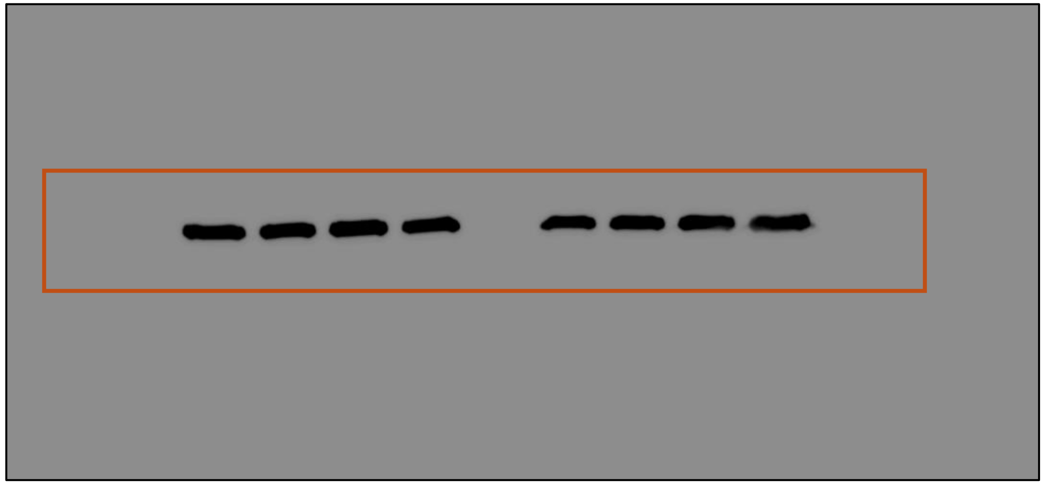

Supplement: Unedited blot and gel images [file jci-135-187778-s147.pdf]
